# Comparative efficacy of violet light, bleaching gel, or their combination, in in-office bleaching: a systematic review and network meta-analysis

**DOI:** 10.1590/1807-3107bor-2026.vol40.056

**Published:** 2026-08-14

**Authors:** Michael Willian FAVORETO, Renata Maria Oleniki TERRA, Camila Mendes CAMARGO, Bianca MARAN, Vanessa CAVALLI, Ana Cláudia CHIBINSKI, Alessandro Dourado LOGUERCIO, Alessandra REIS

**Affiliations:** (a) Universidade de São Paulo - FOUSP, School of Dentistry, Department of Restorative Dentistry, São Paulo, SP, Brazil.; (b) Universidade Estadual de Ponta Grossa - UEPG, School of Dentistry, Department of Restorative Dentistry, Ponta Grossa, PR, Brazil.; (c) Universidade Estadual do Oeste do Paraná - Unioeste, School of Dentistry, Department of Restorative Dentistry, Cascavel, PR, Brazil.; (d) Universidade de Campinas - FOP, School of Dentistry, Department of Restorative Dentistry, Piracicaba, SP, Brazil.

**Keywords:** Tooth Bleaching, Tooth Discoloration, Systematic Review

## Abstract

This systematic review and network meta-analysis aimed to determine whether the use of violet light alone (VIOL) or its combination with bleaching gels (BGVIOL) results in superior bleaching efficacy compared to bleaching gel alone (BG). Randomized clinical trials (RCTs) were identified through searches in PubMed, Cochrane Central, LILACS/BBO, SCOPUS, Web of Science, EMBASE, and gray literature sources in March, 2025. Eligible RCTs compared in-office bleaching using BG, or VIOL, or BGVIOL. The Cochrane risk of bias tool was used to evaluate the risk of bias (RoB 2.0) of the eligible RCTs. A random-effects Bayesian mixed treatment comparison model evaluated color change (ΔE*_ab_ and ΔSGU/final SGU), and risk and intensity of tooth sensitivity (TS). Ranking probabilities were evaluated using the surface under the cumulative ranking curve, and the certainty of evidence was appraised following the Grading of Recommendations Assessment, Development, and Evaluation approach. Out of 4,963 records, 17 RCTs were included, 11 of which had a high RoB. Analysis of ΔE*_ab_ revealed significant mean differences (MD) and 95% credibility interval (95%Crl) for the full dataset for BGVIOL vs. BG (MD = 3.13; 95%CrI: 1.33 to 4.97), but not with the subset of data that excluded studies with high RoB (MD = 2.36; 95%CrI: -0.74 to 5.34). Color change in ΔSGU did not differ among groups. VIOL showed the lowest risk and intensity of TS. Color change achieved with BG and BGVIOL was higher than that obtained with VIOL. VIOL yielded lower risk and intensity of TS. The certainty of the evidence supporting these findings is low or very low.

## Introduction

In-office dental bleaching has gained popularity among patients looking for quick aesthetic solutions, given that some degree of whitening is observed after just one clinical session.^1, 2^ Generally, in-office dental bleaching involves applying the bleaching agent to the tooth surface after retracting the mucosa and soft tissues with lip retractors and protecting the gingival tissues with a light-cured barrier.^1,3^ In most cases, in-office bleaching is carried out using high concentrations of hydrogen peroxide (HP, 30–40%).^4^ Nevertheless, medium (15–25%) and low HP concentrations (6–10%)^4^ and high concentrations of carbamide peroxide (35–37%)^5^ can also be used for in-office bleaching.

The whitening effect occurs through the oxidation of dental organic components by HP and its free radicals.^6^ Although 35% HP alone efficiently bleaches teeth,^7^ light-activation protocols were introduced to provide faster and more effective bleaching based on the fact that light can accelerate the breakdown of HP into free radicals.^8^ Systematic reviews, however, found no clinical advantage in using light-activating devices such as light emitting diodes (LEDs), halogen lights, plasma, or light amplification by stimulated emission of radiation (lasers) associated with in-office bleaching gels.^9, 10^

Although the advantages of light-activated bleaching remain unproven, manufacturers continue developing new devices. Recently, a device emitting violet light at 405–410 nm has been introduced, purportedly matching the peak absorption of potential chromophores.^11^ While these chromophores have not yet been identified in the tooth structure,^12^ their existence has been proposed. As violet light can theoretically degrade these chromophores through physical action,^13^ the use of violet light alone has recently begun to be recommended for dental bleaching.^11^

Violet light has a largely superficial impact, with 98% of violet light intensity attenuated within just one millimeter of enamel,^14^ and despite the relatively low probability of violet light enhancing bleaching efficacy,^9,10^ a growing number of randomized controlled trials (RCTs)^15-24^ are investigating its potential. These studies focus on three key comparisons: A) violet light alone vs. bleaching gel alone,^23,25^ b) violet light alone vs. bleaching gel activated by violet light,^26,27^ c) bleaching gel activated by violet light vs. bleaching gel alone,^2,20,24,28^ and (4) all of the above.^15,17,18,21,22,29-32^ Although these publications enhance our understanding of the efficacy of violet light in bleaching procedures, they have not been appraised for their risk of bias and do not establish the best treatment option among the three evaluated approaches.

Previous systematic reviews on violet light in dental bleaching have important limitations, underscoring the need for a new analysis. Rossi et al.^33^ included a heterogeneous set of studies (*in vitro* experiments, case reports, and only four clinical trials) which compromised the robustness of the evidence and precluded meta-analysis. Similarly, Bessa et al.^34^ conducted a systematic review focused on comparing carbamide peroxide alone compared with its use in combination with violet light. Only three clinical trials, however, met the inclusion criteria, restricting the strength of the conclusions. The main limitation of these reviews was the insufficient number of clinical studies included, which hindered broader comparisons and a more reliable synthesis of the evidence. To bridge this gap, the present study applied a network meta-analysis (NMA), enabling the integration of both direct and indirect comparisons across a larger body of RCTs.

Therefore, the objective of this systematic review was to investigate the bleaching efficacy in ∆E*_ab_ of in-office bleaching protocols (violet light source alone [VIOL], either combined with a bleaching gel [BGVIOL] or bleaching gel alone [BG]) in patients with permanent dentition and assess the relative ranking of these products for each outcome. Other secondary outcomes were also evaluated such as bleaching efficacy in ∆SGU/final SGU, and risk and intensity of tooth sensitivity (TS).

## Methods

### Protocol and registration

This study protocol was registered with the International Prospective Register of Systematic Reviews (PROSPERO – CRD42023433265) and followed the Preferred Reporting Items for Systematic Reviews (PRISMA-NMA) guidelines, with the extension for NMA.^35^

### Eligibility criteria

We included only RCTs with parallel, split-mouth, or crossover designs that evaluated the use of a violet light source for in-office bleaching in permanent dentition. RCTs were excluded if a) bleaching was combined with desensitizing treatments in different groups, b) violet light was compared to another type of light, c) at-home, or d) over-the-counter products were evaluated.

### Information sources and search strategy

The search strategy combined controlled vocabulary (MeSH and Entree terms) and free keywords with the Boolean operators “OR” and “AND”, using the concepts of population (adult patients with permanent dentition) and intervention (violet light-activated in-office dental bleaching).

The MEDLINE search strategy was adapted for use in other electronic databases (Cochrane Library, Brazilian Library in Dentistry, Latin American and Caribbean Health Sciences Literature, Embase, and citation databases [Scopus and Web of Science]). The first search took place on June 27, 2023, and was updated on March 15, 2025. The complete search strategies for all databases are provided in Supplemental Table 1. Supplemental material is available on the Open Science Framework (OSF) platform (https://osf.io/rf5dm/overview). In addition, we reviewed the gray literature by searching abstracts from the International Association for Dental Research and its regional divisions, the System for Information on Grey Literature in the Europe database, and dissertations and theses using the ProQuest Dissertations and Theses full-text database and the Periódicos Capes Theses database.

We also consulted the first 10 pages of Google Scholar (Supplemental Table 1). Ongoing studies were searched in clinical trial registries such as Current Controlled Trials, International Clinical Trials Registry Platform, ClinicalTrials.gov, Brazilian Registry of Clinical Trials, and European Union Clinical Trials Register.

Additionally, the reference lists of the eligible studies were manually inspected for additional relevant publications. In the PubMed database, we also reviewed the first two pages of the related articles linked to each primary study. We did not apply any language restrictions during the search process. Date restrictions were implemented to focus on the most recent violet light units for bleaching. This new violet light source is a visible light produced by four violet LED emitters with a wavelength of 405–410 nm.^14^ To the best of our knowledge, the earliest study involving violet light activation was published in 2011.^36^ To avoid missing any potential earlier prototypes or pilot studies, we extended the search window six years prior to 2011 and included studies published from 2005 onwards.

### Study selection and data collection process

The retrieved references from all databases were stored in a reference management program (EndNote X9, Clarivate Analytics, Philadelphia, USA). Duplicates were removed both automatically using the software and manually by sorting article titles alphabetically. Studies were then screened for relevance in three stages: title, abstract, and full text, using the web-based software Rayyan (Qatar Computing Research Institute, HBKU, Doha, Qatar). Any studies with uncertain eligibility were maintained for the next phase. All phases were carried out by two independent reviewers (M.W.F. and C.M.C.).

Each eligible article received a study identification, combining the first author and year of publication. The same two reviewers summarized and categorized data, such as study design, setting where the study was conducted, mean age, number of participants, baseline color, and tooth on which color measurements were conducted, description of the groups and materials, details of the bleaching protocol (number of sessions, application time, interval between sessions) and details about the light protocol (wavelength, light irradiance, power output), as well as details about the study outcomes (color evaluation tools, TS, and follow-up period). Disagreements were solved by consensus or by consulting a third reviewer (A.R.). If there were multiple reports of the same study (i.e., reports with different follow-ups), data of all reports were extracted directly into a single data-collection form to prevent multiple data entries, following the eligibility criteria.

### Data items and outcomes

All efforts were made to extract data from the primary studies. When graphical results were presented, data were extracted through a specific software (GetData Graph Digitizer version 2.24). When data were missing or presented in a format that did not allow for their extraction, we contacted the authors at most three times, whenever necessary.

When more than one comparison was present in the eligible studies, each comparison contributed a separate entry to the meta-analysis. If numerical outcome data such as the mean or standard deviation (SD) were not available, but sufficient descriptive statistics or alternative summary measures were provided, we derived the missing data using the recommended formulae in the Cochrane Handbook for Systematic Reviews of Interventions^37, 38^ to standardize effect size and maintain methodological consistency. Because the distribution of color change outcomes in bleaching studies is generally symmetric and the mean and median values are typically very close, we considered the median to be an acceptable estimate of the mean when only medians and interquartile ranges were reported.^37, 38^ In these cases, we calculated the SD from the interquartile range using the formulae recommended in the Cochrane Handbook. When a study provided standard errors, confidence intervals or p-values, SD was obtained using the recommended formulae in the Cochrane Handbook for Systematic Reviews of Interventions.^37^

If none of the above methods enabled the calculation of SD from the trial report, the SD was imputed to facilitate inclusion in the meta-analysis, using the average of the coefficient of variation (CV) of the other eligible studies. The study mean was multiplied by the average CV to calculate the missing SD. We recognize the limitations of this approach, particularly its assumption that variability is proportionally similar across studies, which may not always hold true. To address this limitation, we conducted sensitivity analyses to assess the robustness of our results under different imputation strategies.

Some decisions were necessary to enable the meta-analysis. Some studies included more than one group of interest using different products; in such cases, we consistently extracted comparisons involving equivalent gels. For example, in a study that evaluated the groups HP6%; HP35%; and HP6%+VIOL, we only extracted data from the HP6% and HP6%+VIOL groups. This approach ensured that the groups differed only in the presence or absence of light, allowing light to be the sole variable under investigation. In cases in which comparisons involved BG and BGVIOL with two different bleaching agent concentrations, we consistently selected the higher concentration, as they are the most widely used in in-office techniques. For instance, when one study investigated HP35%, HP35%+VIOL, and CP37%+VIO, we included HP35% with and without violet light. Hydrogen peroxide was chosen because it allowed for a direct comparison with and without light, whereas carbamide peroxide was not tested without light. When the number of participants was described in the materials and methods section of the primary articles and a different number was presented in the result tables, we used the data from the results tables, assuming they reflected more closely the number of participants included in the analysis.

Data on color change were recorded as close as possible to 30 days post-bleaching for all analyses. Considering variations in assessment periods reported in the primary studies, the extracted data ranged from seven to 30 days post-bleaching. This decision does not cause clinical heterogeneity because tooth color remains stable for months after bleaching, with no noticeable differences.^39^ After completion of the bleaching protocol, color change in ΔE*_ab_ was measured using spectrophotometers, and color change in ΔSGU was assessed with the Vita Classical shade guide. If ΔSGU was not provided but the final values were available, we extracted the final SGU, as the difference in mean final values is on average the same as the difference in mean change scores.^37^ When studies measured ΔSGU using a spectrophotometer, we combined these data with those that visually assessed ΔSGU using Vita Classical shade guide. If the study, however, presented the instrumental and visual evaluation of the ΔSGU, we extracted the data from the visual evaluation, as this method reflects the traditional clinical method for color matching. If a study employed a split-mouth design, which is rare, given the use of light in the interventions, we included it in the analysis as if it were a parallel-group trial. This decision was further evaluated through a sensitivity analysis to confirm that treating these studies as parallel did not affect the results.

When studies evaluated multiple teeth, data were collected in the following order of priority: canines, central incisors, and other teeth. Due to possible dehydration effects resulting from the bleaching treatment, we prioritized collections at least 48 hours after the in-office procedure.^40^

The absolute risk and intensity of TS reported by the patient during or soon after dental bleaching were assessed. The intensity of TS is commonly measured quantitatively using pain scales, with the visual analog scale (VAS, 0–10) being the most commonly used. Therefore, data from TS was summarized on a 0-10 VAS scale. When the study reported more than one pain scale, we selected the 0-10 VAS. If the study employed another pain scale, data were converted to the 0–10 metric. The absolute risk was measured as the presence or absence of TS. To assess the intensity of TS, both mean and SD were extracted. When intensity was reported across multiple periods, the worst-case scenario was extracted, independently of the treatment week, as data from previous studies did not demonstrate that TS tends to worse over time.^7, 41^ Averaging the data would attenuate peak intensity and could favor certain techniques, not capturing the true patient experience. Reporting a mean TS of “2” if the patient experienced a TS intensity of “6” at any point does not seem to be clinically representative. A single high peak of pain is likely to be recalled by the patient as a painful procedure. Quantitative data were not extracted from studies when they reported a) stimuli-induced TS; b) overall TS across the groups, and c) TS measured using instrumental procedures (not based on patient’s subjective experience). Although some studies also reported stimuli-induced TS, we did not evaluate this outcome as it does not represent clinically spontaneous responses.

When data between information sources were discrepant within the same article, data extraction was prioritized in the following order: a) published article, b) thesis, and c) conference abstracts. This hierarchy reflects the assumption that peer-reviewed published articles typically provide the most reliable and comprehensive information. This prioritization is based on the premise that, although peer review does not guarantee accuracy in all cases, it remains the most widely recognized and effective method to safeguard the quality, transparency, and completeness of scientific reporting.

### Risk of bias (RoB) in individual studies

Each study outcome was independently assessed by two researchers (M.W.F. and C.M.C.) using the Cochrane risk of bias tool (RoB 2.0) for RCTs.^37,42^ This tool evaluated biases from the randomization process (D1), deviations from intended interventions (D2), missing outcome data (D3), outcome measurement (D4), and selective reporting (D5), leading to an overall RoB (OVERALL). Each domain was classified as ‘low RoB,’ ‘some concerns,’ or ‘high RoB’ based on signaling questions. Studies were considered to have low RoB if all domains were rated as low risk, as some concerns if at least one domain raised concerns, and as high RoB if at least one domain was high risk or if two or more domains raised some concerns. Disagreements were resolved through discussion or, if necessary, consultation with a third reviewer (A.R.).

### Network geometry

In the network geometry plot, each node represented a bleaching protocol, with direct comparisons between interventions indicated by lines connecting the nodes. The size of each node was proportional to the number of participants, while the thickness of the connecting lines reflected the number of studies included in each pairwise comparison. The network geometry was visually assessed to evaluate direct pairwise comparisons, evaluate the network structure, and identify any intervention comparisons not linked to the network.

### Summary measures and synthesis of the results

The mean difference (MD) with 95% credible intervals (CrIs) were calculated for the continuous data from the eligible studies (ΔE*_ab_, ΔSGU/final SGU, and intensity of TS). Additionally, the risk ratio (RR) with 95% CrIs was calculated for the dichotomous outcome of TS risk. The meta-analysis was conducted with the studies that reported outcomes in a way that could be extracted, calculated, or converted in the appropriate format for data analysis. Disagreements regarding data extraction were resolved by discussion and consensus.

### Statistical analysis

Initially, a traditional meta-analysis was performed for each pairwise comparison in which evidence was available for two or more studies. Subgroup analysis was performed for studies with low/unclear risk of bias and for those with high risk of bias. Heterogeneity was assessed by using the Cochran Q test and I^2^ statistics. The patient was considered the unit of analysis for all outcomes, as this is standard practice in bleaching trials.

Subsequently, an NMA was conducted using a Bayesian approach to compare treatments both directly and indirectly.^43^ A random effects model was applied, assuming a high likelihood of heterogeneity due to the inclusion of studies from the literature. The NMA was conducted using MetaInsight (version 6.2.0),^44^ which utilizes R packages to conduct network meta-analysis via Bayesian methods with Markov chain Monte Carlo simulations to estimate model parameter distributions. The web tool applies non-informative priors for treatment effects and uses default settings, with 5,000 burn-in iterations and a total of 20,000 iterations (5,001:25,000), generating 20,000 data points per chain. As per the MetaInsight guide,^44^ convergence was assessed using default parameters. After running the dataset, Gelman convergence assessment plots for each mixed treatment comparison were visually inspected, ensuring the potential scale reduction factor (Rc) remained below 1.1, confirming convergence.

NMA results were displayed as point estimates and 95% CrIs. When significant differences were detected between comparisons, we calculated the relative ranking for each intervention using the Surface Under the Cumulative Ranking curve (SUCRA), estimated within the Bayesian framework. SUCRA values range from 0% to 100%, where 100% indicates that the treatment is most likely to be the best option among those compared, and 0% indicates that it is most likely to be the least effective. This approach provides a quantitative summary of the hierarchy of treatments based on the available evidence. Ideally, SUCRA values should be accompanied by the 95% CrI, but these values are not provided yet by the MetaInsight web-tool. Therefore, SUCRA values should be interpreted alongside with differences obtained in the NMA.

Furthermore, sensitivity analyses were conducted to explore the impact of potentially important effect modifiers on NMA findings. These included separate analyses that involved exclusion of the studies with: (1) high RoB and (2) missing data or imputed values.

### Assessment of inconsistency

Inconsistency was assessed locally using the node-splitting method, in which direct and indirect evidence is contrasted (p > 0.05). A statistically non-significant difference between the direct and indirect effects for a split node (p ≥ 0.05) indicates weak evidence to conclude in favor of or against consistency (null hypothesis); otherwise, inconsistency was statistically significant. The weighting of indirect comparisons is based on the precision of the contributing studies and the network structure, so that comparisons supported by larger or more precise trials can contribute more heavily to the estimates.

Additionally, a global assessment of inconsistency was conducted. As MetaInsight does not support running the unrelated mean effects (UME) model or a saturated model for evaluating model fit via Deviance Information Criterion (DIC) comparison, we assessed model consistency indirectly. Leverage plots were examined to identify any data points exceeding the threshold of c = 3 in the consistent model. We also examined the residual deviance from both the NMA consistency model and the UME inconsistency model. This plot illustrates the contribution of each data point to residual deviance in the consistency model (horizontal axis) and the UME inconsistency model (vertical axis). Ideally, most data points should cluster around 1 on the x axis, indicating that consistency is maintained. Additionally, we checked whether the effective number of parameters (pD) was lower than the total number of data points to assess model complexity.

We acknowledge that these methods provide only an indirect assessment of inconsistency and do not fully replace formal inconsistency testing using UME model comparisons or node-splitting approaches. Future developments of the web-tool will allow for these formal assessments and further strengthen the findings.

If local or global inconsistency was detected, we followed a systematic approach to address it. First, we reviewed data extraction and input to identify any errors or misused data. Subsequently, we analyzed study characteristics to determine whether variations in populations, interventions, or outcome definitions could explain heterogeneity. Additionally, subgroup and sensitivity analyses were conducted to assess whether removing poorly fitting studies improved consistency. We also examined the network structure to detect any unusual treatment connections. Finally, we refitted the models in R, testing alternative model assumptions to evaluate their impact on inconsistency. If no clear cause was identified or if an article contributing to inconsistency was found, we proceeded with a consistent model while transparently acknowledging this limitation in the revised manuscript.

### Assessment of the certainty of evidence using the grading of recommendations: assessment, development, and evaluation

The certainty of evidence for each comparison and outcome was assessed following the guidance suggested by the Grading of Recommendations Assessment, Development and Evaluation (GRADE) Working Group.^45,46^ First, we assessed certainty of evidence in direct estimates based on the classic GRADE domains, including risk of bias, inconsistency, indirectness, and publication bias. Subsequently, the certainty of indirect estimates was rated by identifying the first-order loop with the lowest certainty and considering potential intransitivity. Lastly, the certainty in network estimates was rated based on the higher certainty between the direct and indirect estimates, while also accounting for incoherence and imprecision at the network estimate level. To assess the imprecision domain, we employed a minimally contextualized approach, using the minimally important difference (MID) as the threshold.^47,48^

Judgments regarding MID effects were based on published minimally important differences and consensus among the authors. We adopted a conservative MID threshold of 1 point for NRS scale and 2 points for VAS 0-10 for intensity of TS, while setting it at 100 per 1,000 patients for the risk of TS.^48^ For color change, we used the established acceptability threshold (ΔE*_ab_ = 2.7)^49^ for instrumental evaluation and a 2.0 shade guide unit difference for visual assessment, based on the published literature.^50^.We reported the results using GRADE evidence tables with detailed explanatory footnotes.^45,51^

## Results

### Study selection

**Figure 1:**
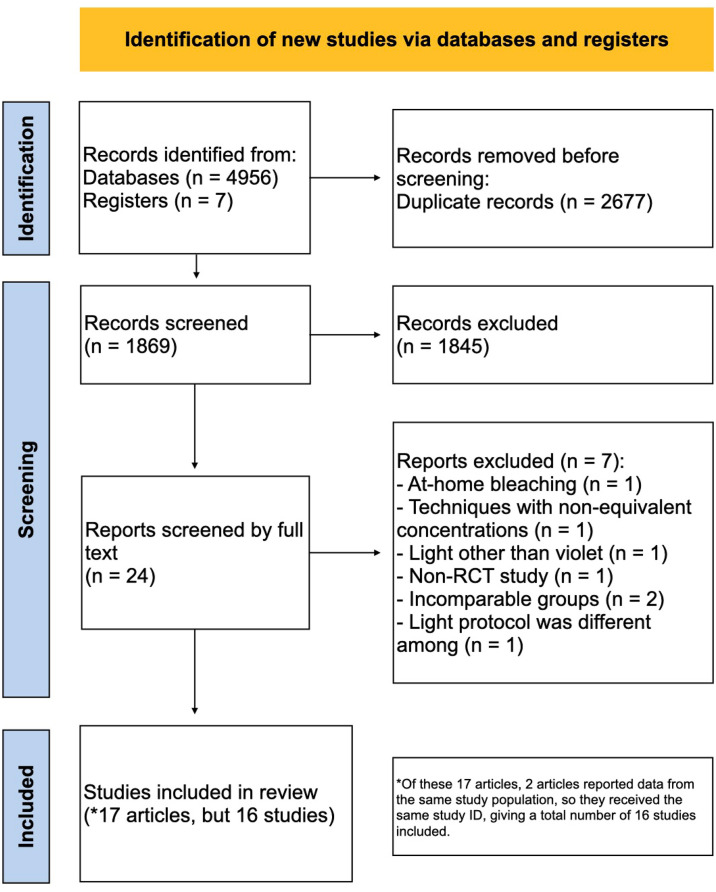
PRISMA 2020 flow diagram illustrating the identification, screening, eligibility assessment, and inclusion of studies in the systematic review.

A total of 4,963 articles were retrieved from electronic databases. After the removal of duplicates, and title and abstract screening, 24 articles remained (Figure 1). Of these articles, seven were excluded for various reasons: at-home bleaching;^19^ techniques with non-equivalent concentrations;^52^ light other than VIOL;^53^ non-RCT study;^54^ incomparable groups;^16,55^ and differences in light protocols.^56^ A total of 17 articles remained for the systematic review.^15 17 18 20-32^ Of these 17 articles, two^15 18^ reported data from the same study population, so they received the same study ID, totaling 16 included studies.

RCT: randomized controlled trial. The search was conducted on June 27, 2023, and updated on March 15, 2025.

### Study characteristics

The characteristics of the eligible RCTs are presented in Supplemental Table 2. Studies with a parallel design (n = 15) were the most common, and only one study used the split-mouth design;^2^ therefore, its inclusion as a parallel study had a negligible impact. All studies evaluated color change either using visual methods (n = 11) or instrumental methods (n = 15), or both (n = 8). Among the studies that used instrumental evaluation, 14 used a spectrophotometer and one used a colorimeter. Regarding visual evaluation, 11 studies employed the Vita Classical Scale/Vitapan Classical Scale. Four studies^20, 21, 28, 30^ assessed ΔSGU or final SGU using a spectrophotometer. Two studies evaluated ΔSGU visually and instrumentally; in this case, we used the data from visual inspection.^20,28^

All studies evaluated TS using the VAS scale. Fourteen studies evaluated the risk of TS and 12 assessed the intensity of spontaneous TS. One study assessed risk and intensity but selectively reported data for only one group, and the data could not be used.^17^ Two studies evaluated stimuli-induced and spontaneous TS.^24,28^ Two studies evaluated only stimuli-induced TS.^23, 27^ Only spontaneous TS data were used for the analyses.

Nine studies did not report the participant’s age. In the other studies, included participants were aged 18 to 60 years, with a mean of 23 years, showing a predominance of young adults. Women were predominant in all studies reporting gender.

Regarding the bleaching protocol, HP gels were the most used (n = 14 studies) either at low or high concentrations. Carbamide peroxide gels (35–37%) were used in four RCTs. A single 30-minute session was the most widely used protocol (Supplemental Table 2). The characteristics of the groups, protocols, and assessment methods of the eligible articles were extracted and described only for the groups used in this review, as specified in the ‘Data items and outcomes - Decision-Making data items and outcomes section.

Only two violet light devices were employed. The Bright Max Whitening BMW (MMOptics, São Carlos, SP, Brazil) was employed in 14 studies, while the Whitening Plus device (DMC, São Carlos, SP, Brazil) was used in two studies.^24, 28^ The most commonly used light application protocol consisted of 20 one-minute applications, with 20-second intervals between the applications.

### Risk of bias within and across studies

**Figure 2:**
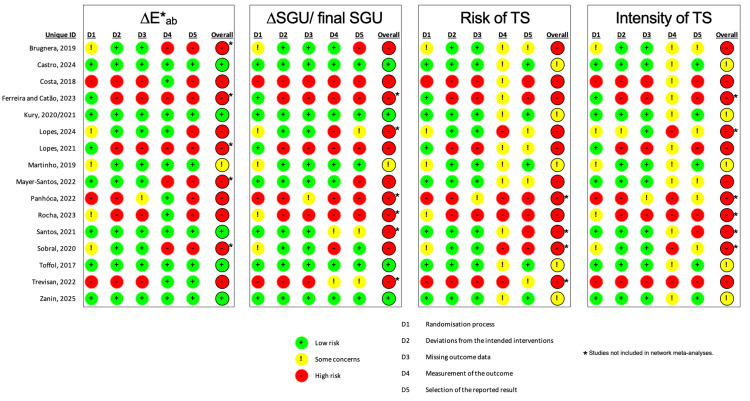
Summary of risk of bias assessment (RoB 2.0, Cochrane Collaboration) for bleaching efficacy in ∆E*ab and ∆SGU/final SGU, as well as for risk and intensity of tooth sensitivity (TS).

In the evaluation of ∆E*_ab_, five out of 16 studies exhibited a low RoB, one had some concerns, and 10 showed a high RoB (Figure 2). For ∆SGU/final SGU, four studies had a low RoB, one had some concerns, and 11 had a high RoB. Regarding the risk of TS, none of the studies had a low RoB, while five showed some concerns and 11 were considered high risk. Similarly, for TS intensity, no studies were rated as low risk, five raised some concerns, and 11 exhibited a high RoB (Figure 2).

The risk of bias was evaluated across five domains: D1 (randomisation process), D2 (deviations from the intended interventions), D3 (missing outcome data), D4 (measurement of the outcome), and D5 (selection of the reported result). Each study is categorized as low risk (green), some concerns (yellow), or high risk (red). Studies marked with (*) were not included in network meta-analyses as data were not reported or were assessed in another way that is not clinically relevant.

### Network meta-analyses

Given the high percentage of studies with a high risk of bias (RoB) across all outcomes, the network meta-analysis was conducted using all eligible studies, alongside a sensitivity analysis that excluded those with high RoB. This comparative approach provides a more robust assessment of the impact of RoB on the findings. Unless the sensitivity analysis yielded different conclusions, results presented in this section reflect the full dataset. Imputation was required in only two studies for objective color analysis^17, 24^ and in one study for subjective color analysis.^22^

### Instrumental color change in ∆E*ab

**Figure 3:**
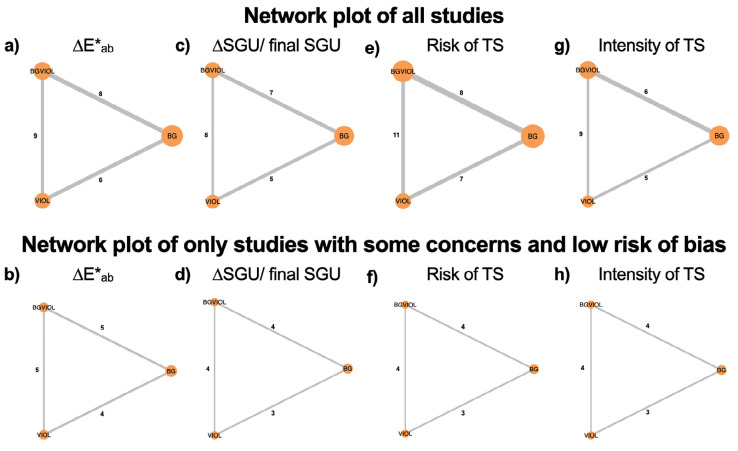
Network plot geometry for bleaching efficacy in ∆E*ab and ∆SGU/final SGU, as well as for risk and intensity of tooth sensitivity (TS).

VIOL: violet light only; BGVIOL: bleaching gel combined with violet light; BG: bleaching gel only*.* The upper row represents all included studies, while the lower row includes only studies with some concerns and low risk of bias. Nodes represent the different interventions, and edges indicate direct comparisons between them, with thicker lines representing a higher number of studies included in each comparison.

The full dataset included 11 eligible studies with a total of 506 patients (Figure 3a). After excluding studies with a high RoB (Figure 3b), six studies remained, with a total of 360 patients. In both analyses, the network geometry formed a closed triangular loop. In the network geometry, line thickness represents the number of RCTs in each pairwise comparison, and the node size reflects the relative number of participants in each treatment group (Figure 3).

**Figure 4:**
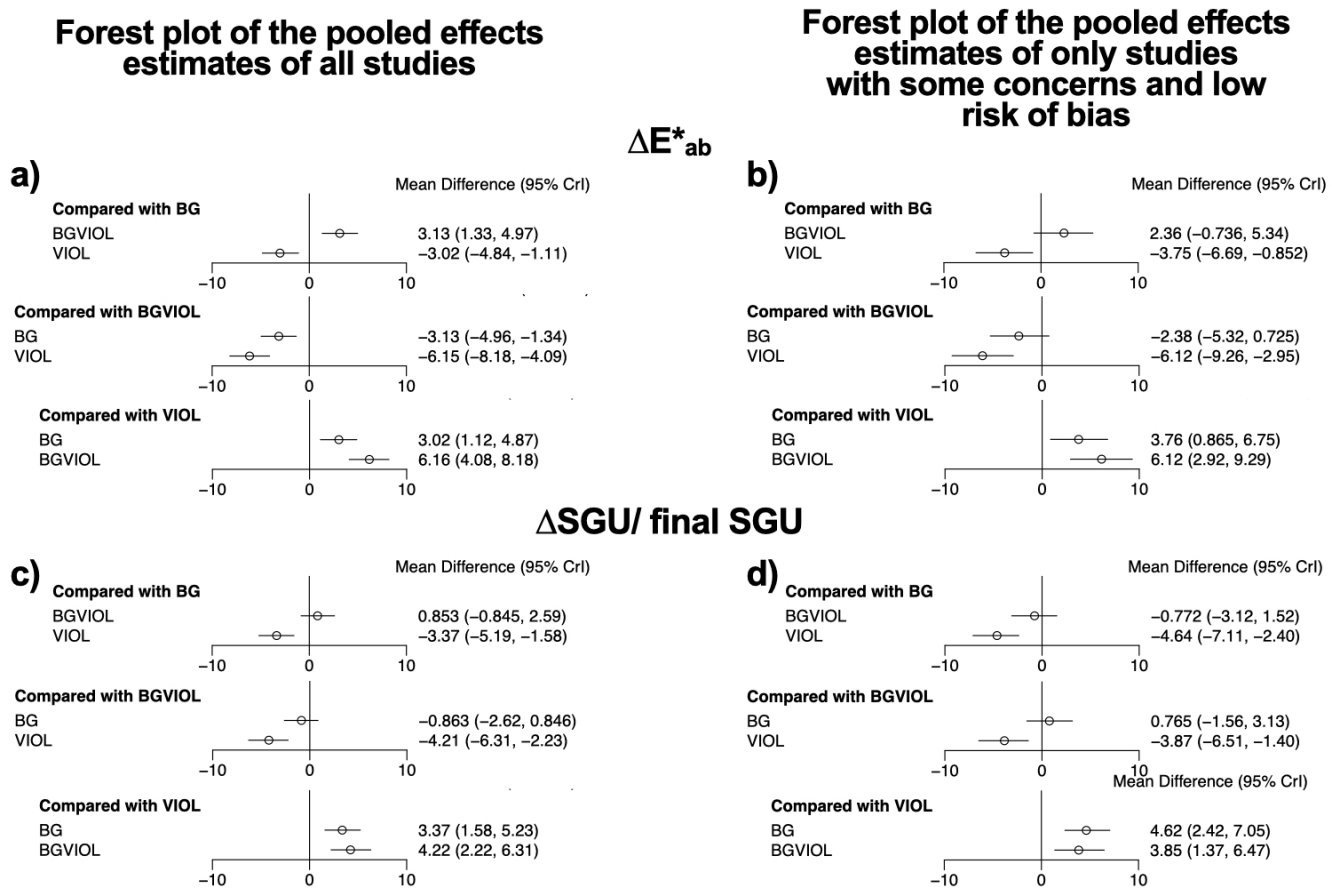
Forest plot of the pooled effects estimates for color change in ∆E*ab and ∆SGU/final SGU, comparing different bleaching protocols.

VIOL: violet light only; BGVIOL: bleaching gel combined with violet light; BG: bleaching gel only The left side represents all included studies, while the right side includes only studies with some concerns and low risk of bias. Data are expressed as mean difference and respective 95% CrI. Comparisons were made between BGVIOL and BG, VIOL and BG, and VIOL and BGVIOL.

In the NMA that included all studies, five two-arm trials compared VIOL vs. BG^25,27^ and BGVIOL vs. BG^2,24,26^, while six studies were three-arm comparisons of all three interventions^17,18,29-32^. Prior to conducting the NMA, traditional random-effects pairwise meta-analyses with subgroup analyses for RoB were performed, and the results are presented in Supplemental Figure 1. Bayesian random-effects forest plots summarize the pooled effect estimates for ΔE*_ab_ as mean difference [95%CrI] for all eligible studies (Figure 4a) and for those with low or unclear RoB (Figure 4b).

The VIOL group demonstrated statistically inferior color change compared to BG [MD = -3.0; 95%CrI -4.8 to -1.1] and BGVIOL [MD = -6.2; 95%CrI -8.2 to -4.1]. While BGVIOL initially appeared to produce greater color change than BG in the NMA that included all studies (Figure 4a), this effect was no longer observed when high-RoB studies were excluded [MD = 2.4; 95%CrI -0.7 to 5.34; Figure 4b], suggesting that high-RoB studies inflated the results in favor of BGVIOL.

### Visual color change evaluation in ∆SGU/final SGU

Two NMAs were conducted: (a) including all 10 eligible studies evaluating three interventions in a total of 622 patients, and (b) excluding studies with a high RoB (five studies, 360 patients). In both Bayesian NMAs (Figs. 3b and 3f), the network geometry formed a closed triangular loop. In the NMA including all studies (Figure 3b), five two-arm trials compared VIOL vs. BG^23,25^ and BGVIOL vs. BG^2,20,28^, while five three-arm studies evaluated all three interventions^18,22,29,30,32^.

Prior to conducting the NMA, traditional random-effects pairwise meta-analyses with subgroup analyses for RoB were performed, with results presented in Supplemental Figure 2. Bayesian random-effects forest plots summarize the pooled effect estimates for ΔSGU/final SGU as MD [95% CrI] for all eligible studies (Figure 4c) and for those with low or unclear RoB (Figure 4d). Both analyses found that the VIOL group produced statistically inferior color change to BG [MD = -3.4; 95% CrI -5.2 to -1.6] and BGVIOL [MD = -4.2; 95% CrI -6.3 to -2.2]. BGVIOL and BG showed no significant difference in color change [MD = 0.85; 95% CrI -0.85 to 2.6; Figure 4c] in both full dataset and the dataset that excluded studies with a high RoB (Figure 4d).

### Risk of TS

We conducted two network meta-analyses: a) including all 12 eligible studies, evaluating three different interventions in 682 patients, and b) excluding studies with a high RoB (total of five studies and 300 patients).

For both Bayesian network meta-analyses (Figure 3c and 3g), the network geometry formed a closed triangular loop. In the NMA that included all studies (Figure 3c), five were two-arm trials investigating the comparisons between VIOL and BG ^25^ and between BGVIOL and BG,^2, 20, 26, 28^ and seven studies included all three interventions (VIOL vs. BGVIOL vs. BG).^17, 18, 21, 29-32^

**Figure 5:**
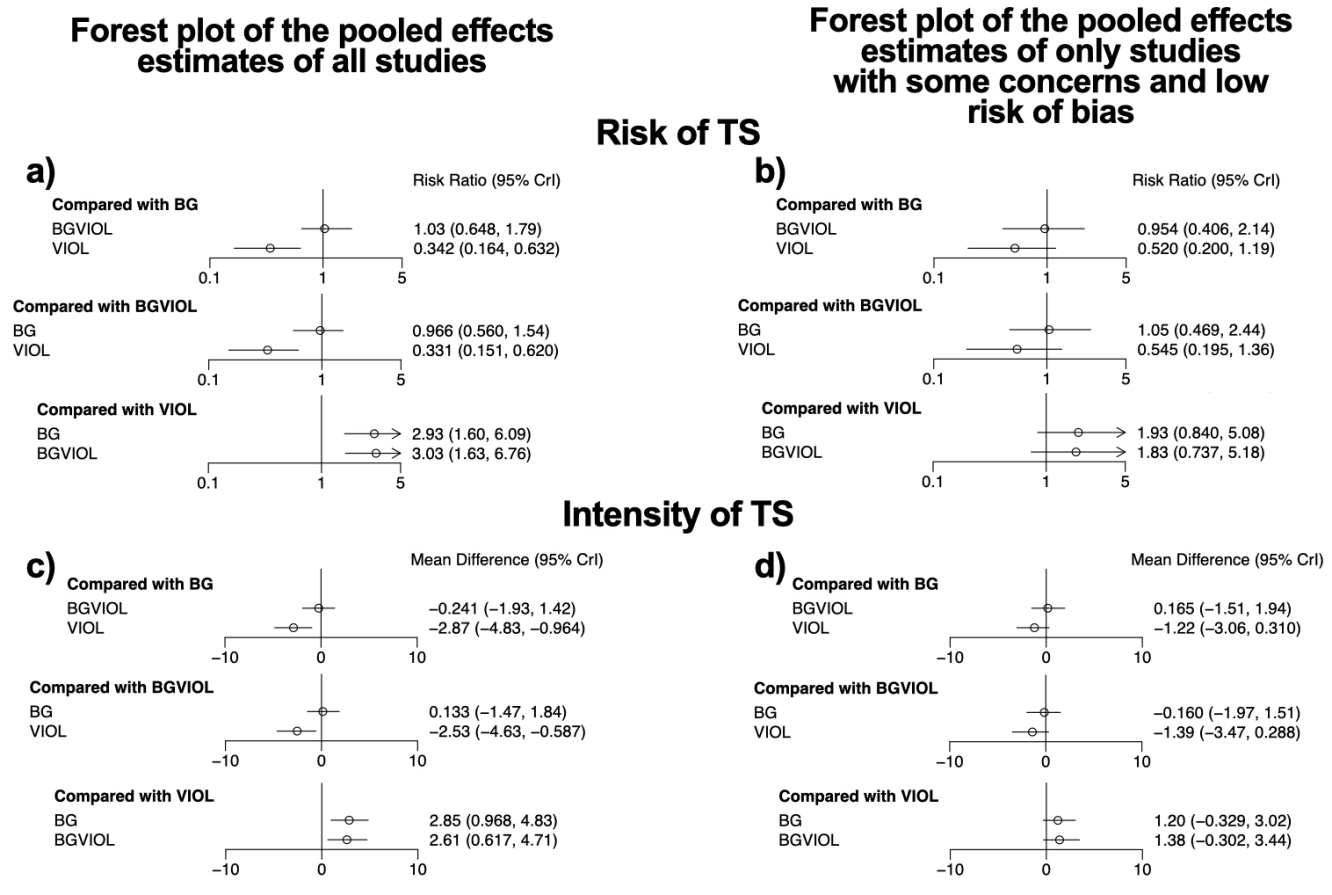
Forest plot of the pooled effects estimates for risk and intensity of tooth sensitivity (TS), comparing different bleaching protocols.

VIOL: violet light only; BGVIOL: bleaching gel combined with violet light; BG: bleaching gel only. The left side represents all included studies, while the right side includes only studies with some concerns and low risk of bias. Data are expressed as risk ratio or mean difference and respective 95%CrI. Comparisons were made between BGVIOL and BG, VIOL and BG, and VIOL and BGVIOL.

Prior to the NMA, traditional random-effects pairwise meta-analyses with subgroups analysis for the risk of bias were conducted and the results can be seen in Supplemental Figure 3. Bayesian random-effects forest plots with the pooled effect estimates for risk of TS are depicted as risk ratios (RR [95% CrI]) for all eligible studies (Figure 5a) and for studies with low or unclear RoB (Figure 5b). The full dataset analysis showed that the VIOL group showed a statistically lower risk of TS than did BG [RR = 0.34; 95% CrI -0.2 to 0.6] and BGVIOL [RR = 0.33; 95% CrI 0.2 to 0.6] (Figure 5a). BGVIOL and BG were statistically similar [MD = 1.0; 95% CrI 0.65 to 1.8; Figure 5a]. Nevertheless, the dataset excluding studies with high RoB did not detect any statistically significant difference in RoB across the three groups, likely due to lower power from the smaller number of included studies (Figure 5b).

### Intensity of TS

The network meta-analysis for the full dataset included 10 eligible studies, evaluating three different interventions in 576 patients, while the NMA excluding studies with a high RoB included five studies with 300 patients.

For both Bayesian NMAs (Figure 3d and 3h), a closed triangular loop was formed. In the NMA with all studies (Figure 3d), five were two-arm trials investigating the comparisons between VIOL and BG ^25^ and between BGVIOL and BG,^2,20,24,28^ and five studies included all three interventions (VIOL vs. BGVIOL vs. BG).^18,22,29,30,32^

Traditional random-effects pairwise meta-analyses with subgroups analysis for the RoB were conducted and the results are depicted in Supplemental Figure 4. Bayesian random-effects forest plots with the pooled effect estimates for intensity of TS as MD [95% CrI] for all eligible studies can be found in Figure 5c, while those studies with low or unclear RoB are shown in Figure 5d. The full dataset analysis showed that BGVIOL vs. BG were statistically similar ( MD=−0.24 [95\%CrI -1.93 to 1.42]  ). The intensity of TS was significantly lower for VIOL either when compared with  BG (MD=−2.87 [95\%CrI -4.83 to -0.964]  , or with  BG+VIOL (MD = -2.63 [95%CrI−4.68 to −0.60] ; Figure 5d).

### Consistency of the data

**Figure 6:**
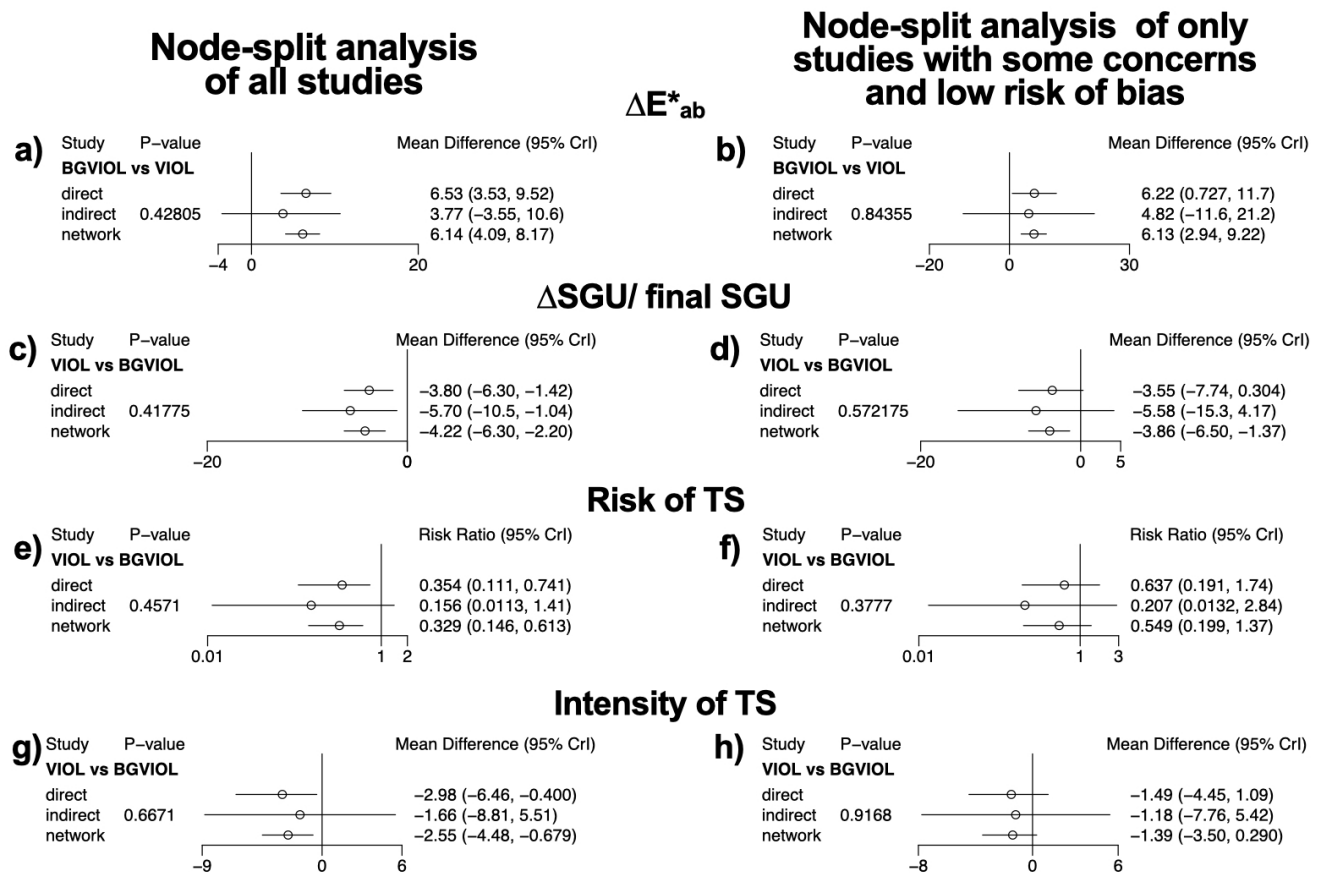
Node-split analysis for color change in ∆E*ab, ∆SGU/final SGU, risk of tooth sensitivity (TS), and intensity of TS, comparing direct and indirect evidence.

Local consistency evaluation (node-split analysis) between direct and indirect evidence was only possible for the BGVIOL and VIOL pair in which indirect and direct evidence was taken from independent sources (Figure 6). We observed overlapping 95% CrIs for applications, with ∆E*_ab_, preventing us to reject the null hypothesis of consistency (p = 0.43). Similarly, the same was observed for ∆SGU/final values (p = 0.41), risk of TS (p = 0.45), and intensity of TS (p = 0.66). These conclusions did not change in the sensitivity analysis that excluded studies with high RoB (Figure 6).

The left panel presents results from all included studies, while the right panel includes only studies with some concerns and low risk of bias. The p-values show the probability under the null hypothesis that both sources of evidence are equal, with p-values lower than 0.05 suggesting inconsistency between the two sources of information.

The leverage plots for the consistent model (Supplemental Figure 5) showed no data points exceeding the threshold of c = 3, supporting the assumption of consistency for DE*_ab_, ΔSGU/final, SGU, and risk of TS. Additionally, we examined the residual deviance from the NMA consistency model (Supplemental Figure 6). Most data points clustered around 1 on the x axis, further indicating that the consistency model was appropriate, except for the intensity of TS for the full dataset. All these analyses were also conducted on studies with low or unclear RoB, yielding the same conclusions.

Residual analysis indicated that most values remained below 2.0, meaning that observed and predicted values aligned well (Supplemental Figure 7) for the consistent model for all network meta-analyses, except for the intensity of TS in the full dataset. As for TS intensity, while most residuals were within an acceptable range, one study^30^ showed an excessively high residual (>5.5), suggesting a disproportionate influence on TS intensity (Supplemental Figure 7). After the exclusion of the study with the excessively high residual from the sensitivity analysis, the model parameters stabilized, leading to better statistical fit and convergence of the MCMC chains (Supplemental Figure 7).

Model fit to a consistent model was further assessed via posterior mean deviance (Dbar), effective pD, total number of points, and DIC (sum of Dbar and pD). (Supplemental Table 3). In all NMAs presented herein, pD was lower than the total number of data points, ensuring low complexity of the model, consistent with consistency model assumptions.

### Convergence of the model

The Gelman-Rubin test for convergence indicated that the potential scale reduction Factor (R) was close to 1 after a burn-in of 5,000 iterations, demonstrating adequate convergence of the MCMC chains for all the network meta-analyses (Supplemental Figure 8). An exception was observed for the intensity of TS. For this outcome, the Gelman-Rubin convergence assessment did not reveal convergence of MCMC chains, suggesting that the estimates might be unreliable. The shrinkage factor did not converge to 1.0, particularly for the BGVIOL vs. BG and BG vs. VIOL comparisons, in which values remained elevated even after 25,000 iterations. The shrinkage factor exceeded 1.2 for key parameters.

### SUCRA rankings

**Figure 7:**
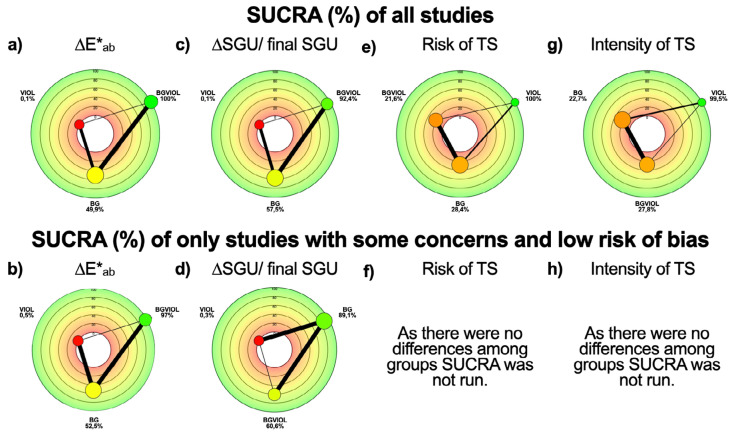
The rank probability (SUCRA) of different interventions for bleaching efficacy in ∆E*ab and ∆SGU/final SGU, as well as for risk and intensity of tooth sensitivity (TS).

VIOL: violet light only; BGVIOL: bleaching gel combined with violet light; BG: bleaching gel only. The upper row represents all included studies, while the lower row includes only studies with some concerns and low risk of bias. SUCRA (%) values indicate the probability of each intervention being ranked as the most effective or least harmful. As there were no differences among groups for risk and intensity of TS in the lower row, SUCRA analysis was not conducted for these outcomes.

SUCRA values were presented for all eligible studies, excluding those with high RoB. For ∆E*_ab_, the VIOL group had the lowest probability of being the most effective for dental bleaching (0.1% for the full dataset and 0.5% for the dataset that excluded studies with a high RoB). Although BGVIOL (100% and 97%) presented higher rankings than BG (49.9% and 52.5%), these figures should not be interpreted as superiority, as BG and BGVIOL did not statistically differ in the network meta-analysis (Figure 7).

The same pattern was observed for Figures 5a and 5e, which presents the SUCRA rankings for ∆SGU/final SGU. The VIOL group had the lowest probability of being the most effective for dental bleaching (0.1% and 0.3%, respectively). For BGVIOL, the SUCRA rankings were 92.4% and 60.6%, respectively, and 57.5% and 89.1% for BG. Again, these figures should not be interpreted as indicating superiority, as BG and BGVIOL did not statistically differ in the network meta-analysis (Figure 7).

For both the risk and intensity of TS, SUCRA values for the full dataset showed that VIOL had the highest probability of causing less risk of TS (100%), while BG (28.4%) and BGVIOL (21.6%) had the lowest probabilities. These probabilities were 99.5%, 22.7%, and 27.8%, respectively, for the intensity of TS. SUCRA analysis was not run for the subset of these two datasets that excluded studies with RoB, as they did not differ statistically in the network meta-analysis (Figure 7).

### Sensitivity analysis

The sensitivity analysis excluding studies with high RoB was presented alongside with all eligible studies. Another sensitivity analysis, excluding studies where in which data needed to be imputed, showed minor absolute variations, but led to the same conclusions as the analysis that included all studies. When data were not imputed, this sensitivity analysis was not conducted (Supplemental Figure 9).

### Certainty of evidence

The quality ratings for direct, indirect, and network evidence for each outcome are provided in the Supplemental Material. Most of the comparison pairs were assessed as having low or very low quality of evidence, mainly due to high risk of bias and serious imprecision (Supplemental Tables 4 through 7).

## Discussion

A major issue in this study was the high number of trials with a high RoB. Many studies inadequately described the randomization process, particularly allocation concealment, which is crucial for ensuring unbiased participant distribution and preventing selection bias. Some studies failed to implement proper randomization, leading to baseline imbalances, while others did not report baseline data. Randomization methods should be clearly described, specifying sequence generation and concealment techniques, such as computerized systems or opaque envelopes.

Blinding (domain 2) was often poorly reported or ambiguous, increasing the risk of deviations from intended interventions. Inadequate blinding was particularly concerning in procedures involving light- or sound-emitting devices, in which placebo and Hawthorne effects could influence patient perception. Strategies such as simulated light (placebo), opaque masks, protective glasses, and simulated sounds can help ensure blinding.

High dropout rates and missing outcome data (domain 3) were common, with unclear reporting on how missing data were handled. Per-protocol analyses, which excluded non-compliant patients, further contributed to attrition bias, reducing statistical power and distorting intervention effects. While intention-to-treat analysis mitigates some of these issues, it is insufficient when dropout rates are excessive.

Evaluator blinding (domain 4) was less critical for instrumental assessments but essential for subjective color evaluation (∆SGU) and TS-related outcomes, for which patient perception plays a key role.

Clinical trial registration is essential for transparency, scientific integrity, and ethics. It ensures that study objectives, methods, and outcomes are publicly available, reducing publication bias and enhancing reproducibility. Registration prevents redundancy, ensures adherence to ethical protocols, and protects participants. Maintaining methodological rigor by following the registered protocol improves data reliability, while any necessary changes should be clearly justified to maintain transparency. A common problem was the lack of protocol registration (Domain 5), which prevented comparisons between reported and planned outcomes. Key outcomes such as bleaching efficacy (∆E*_ab_ and ∆SGU/final SGU) and TS risk/intensity were often not reported, raising concerns of selective reporting. Many studies also failed to account for clustering effects when analyzing multiple teeth from the same patient, and unplanned repeated statistical testing increased the risk of type I errors in these studies.

Altogether, these factors collectively contributed to the low or very low certainty of the evidence, as determined by the GRADE analysis. Consequently, side-by-side NMAs were reported for the full dataset of primary studies and for the subset that excluded studies with a high RoB, allowing for evaluation under both conditions.

The use of light sources to enhance dental bleaching has been extensively investigated for years.^57^ While HP alone is effective, some manufacturers claim that light can shorten the time required to achieve the desired whitening effect.^8^ From a chemical perspective, this claim is theoretically plausible.^8^ Light sources such as LEDs, halogen lights, plasma, or lasers transfer energy directly to HP or to photosensitizers in the bleaching product, intensifying its photochemical degradation into free radicals.^8^ The heat generated by light may further accelerate this process. These free radicals then break down or modify organic compounds in the tooth structure, reducing their ability to absorb certain wavelengths of visible light and increasing light reflection, resulting in a whitening effect. However, despite generating more free radicals, the procedure has not been shown to enhance bleaching efficiency, a finding consistent with previous systematic reviews on this topic.^9,10^

The fact that light can increase free radical production has led manufacturers to introduce various light-based bleaching technologies into the market. The most recent innovation involves violet light with a wavelength of 405–410 nm.^15,17,18,20-32^ This systematic review with NMA is the first to investigate the impact of this device on bleaching efficacy and associated side effects. We evaluated color change using both instrumental analysis (ΔE*_ab_) and visual assessment (ΔSGU/final SGU), considering the full dataset of primary studies and a subset that excluded studies with a high RoB. Except for the full dataset evaluation of ΔE*_ab_, the other three analyses showed no significant difference between bleaching gel alone and bleaching gel combined with violet light. Additionally, across all four analyses - instrumental and visual, using both the full dataset and the subset that excluded studies with a high RoB - we observed that light alone, despite claims of whitening efficacy by some manufacturers, was inferior to both BG and BGVIOL and was not effective for dental bleaching.

Despite the advantage of the BGVIOL group in the full dataset of ΔE*_ab_ (MD = 3.13, 95%CrI 1.33 to 4.97) compared to BG (Figure 4a), the clinical significance of this difference is borderline. Considering that the acceptability threshold for color change (50:50% PT) in ΔE*_ab_ is 2.7,^49^ the observed difference, albeit statistically significant, may not be clinically relevant for all patients. Given the high investment required for acquiring and maintaining violet light-emitting devices, the low certainty of the evidence, and the fact that this difference was observed in only one out of four comparisons, the adoption of this technology remains uncertain and cannot be recommended based on current knowledge. Future studies should assess the cost-benefit ratio, considering not only whitening efficacy but also patient perception and the economic feasibility of the technique.

In aesthetic dentistry, the search for innovation often leads to the rapid adoption of new technologies before strong scientific evidence is available. This may be influenced by novelty bias, in which manufacturers, clinicians, and patients favor a new product simply because it is new, assuming it will perform better. Marketing and the appeal of modern technology can strengthen this belief, even in the absence of robust data. Violet light technologies are a good example: they have become increasingly popular, but scientific evidence is still limited and methodologically inconsistent. This highlights the need for well-designed and sufficiently powered clinical trials that evaluate both objective results and patient-centered outcomes to guide clinical recommendations.

Clinical recommendations should be grounded in evidence with a low risk of bias. Given that such evidence is not yet available for this intervention, the principle of the null hypothesis, assuming no difference between treatments, should be upheld. Until robust, high-quality evidence becomes available, clinicians should refrain from adopting this device in routine practice.

SUCRA is a valuable tool for determining the relative ranking of treatments for a given outcome. MetaInsight software and the gemtc package in R, however, do not provide CrIs for SUCRA values, limiting the ability to assess the uncertainty associated with treatment rankings. Although BGVIOL showed a high SUCRA ranking for color change, it is likely that its 95% CrI overlaps with that of the BG group, preventing definitive conclusions about superiority. Future advancements in MetaInsight and NMA packages should incorporate 95%CrIs for SUCRA rankings to improve the interpretability and reliability of treatment hierarchies.

Objective evaluation is crucial for assessing the efficacy of dental bleaching, as it minimizes the subjective influence of the evaluator. Such evaluation requires spectrophotometers, colorimeters, or calibrated digital cameras, which measure color based on numerical parameters, such as the coordinates of the CIELab space (L*, a*, b*). This approach eliminates reliance on the evaluator and controls external factors, providing greater precision and reproducibility. Nevertheless, the high cost of equipment and the need for technical training to operate it may limit its adoption in clinical settings.

In this systematic review, instrumental color assessment was based on ΔE*_ab_ values derived from CIELab coordinates (L, a*, and b*), measured at different time points in primary studies. More advanced formulae, such as ΔE_00_,^58^ provide greater precision and better correlation with visual perception. However, due to limited data availability, ΔE_00_ could not be incorporated into this review. Another promising metric, the whiteness index for dentistry (WI_D_),^59^ also remains underreported in the literature. RCTs on dental bleaching should adopt these newer color change parameters, including ΔE_00_ and WI_D_, while retaining ΔE*_ab_ to facilitate comparisons between past and present studies.

Subjective evaluation relies on the visual perception of the evaluator, typically using standardized color scales such as Vita Classical. This method is influenced by the observer’s experience and external factors, including lighting conditions, visual fatigue, and viewing angle. Although widely used in clinical practice due to its accessibility and low cost, subjectivity introduces inter- and intra-observer variability, reducing result consistency. Nonetheless, it remains a practical and sufficient approach for most clinical cases. Additionally, it serves as a convenient tool for dentists, who commonly use it in their offices to monitor the progression of dental bleaching. As previously discussed, this study found no significant advantage of BGVIOL over BG in both the full dataset and the subset excluding studies with a high risk of bias. Furthermore, it confirmed that VIOL light alone has very limited efficacy in dental bleaching.

A common practice in the primary studies included in this systematic review was the use of spectrophotometer-derived shade guide units instead of traditional visual evaluation. The conversion method used by these devices, however, is often unclear. Considering that color scales were originally designed for direct visual interpretation, the correlation between objective and subjective methods is not always straightforward. This can create unnecessary complexity and confusion, particularly because most professionals lack access to sophisticated equipment, while traditional shade guides remain effective in daily clinical practice.

Bleaching-induced TS is the most common side effect of dental bleaching.^60^ Hydrogen peroxide travels within minutes to the dental pulp,^6,^ where it triggers an inflammatory reaction, resulting in acute, localized, and short-lasting pain. TS typically subsides within 48 hours after treatment.^7^ In the absence of hydrogen peroxide, such as in the VIOL group, TS does not occur, which clearly explains the lower risk and intensity of TS in the full dataset of primary studies. Excluding studies with high RoB reduced the number of studies and, consequently, statistical power to detect any significant difference among the three groups.

One of the models analyzing TS intensity did not converge. Although no clear explanation could be provided, this finding should be interpreted with caution, as such analyses are typically underpowered and may yield false-positive and negative results. Future updates of this systematic review, including additional RCTs on the topic, will enable a more robust investigation of potential reasons if the lack of convergence persists.

The evaluation of TS in clinical studies should consider its practical relevance. Artificial induction methods, such as tactile, air, or thermal stimuli, do not reflect real clinical conditions and may overestimate TS. Conversely, spontaneous patient reports during their routine provide more accurate and applicable data, better representing the real clinical experience. Moreover, prioritizing outcomes based on self-reports minimizes bias and enhances the relevance of results for dental practice, making this an essential aspect for developing studies more aligned with clinical needs. This is the reason why we only included spontaneous TS data in this systematic review.

This review is limited by the inability to explore potential effect modifiers, such as baseline tooth shade, light intensity, and hydrogen peroxide concentrations. These variables may influence bleaching efficacy and TS risk and intensity whether used with or without light activation, but the limited number of available trials precluded a formal statistical analysis. The impact of these modifiers can only be adequately assessed when a larger body of well-reported RCTs becomes available, allowing for robust subgroup or meta-regression analyses.

Note that the main objective of this NMA was to investigate the specific role of VIOL in in-office bleaching protocols. Although some comparisons included lower concentrations of hydrogen peroxide (e.g., 6%), most of the included studies involved higher concentrations, as they were more frequently reported and allowed for broader network connections. We recognize, however, that the interaction between BGVIOL may vary depending on peroxide concentration. Future studies should further explore the clinical behavior and potential synergistic effects of VIOL when used with lower-concentration bleaching gels, which are increasingly being adopted in clinical settings due to their potential to reduce adverse effects such as TS. We encourage future clinical trials to include more patient-centered outcomes, such as aesthetic satisfaction and perceived discomfort, to enhance the clinical relevance of bleaching research.

## Conclusions

Based on the currently available evidence, VIOL alone does not appear to produce clinically significant tooth bleaching, and its combination with BG does not seem to enhance bleaching efficacy compared with BG alone. These findings were consistent at different peroxide concentrations. However, the certainty of this evidence was rated as low or very low, and results should be interpreted with caution. Well-designed, adequately powered RCTs using comprehensive color assessment parameters (e.g., WI_D_ and ΔE_00_) are strongly recommended to confirm or refute these findings and guide clinical practice.

## Supplementary Materials

Supplementary Figures

Supplementary Tables

## Data Availability

The datasets generated during and/or analyzed during the current study are available from the corresponding author on reasonable request.
