# Supplementary material for: Comparative efficacy of violet light, bleaching gel, or their combination, in in-office bleaching: a systematic review and network meta-analysis
Source: Braz Oral Res. 2026 Aug 14;40:e056. doi: 10.1590/1807-3107bor-2026.vol40.056 (PMC13475733; doi:10.1590/1807-3107bor-2026.vol40.056)

Supplementary Figure 1. Pairwise meta-analysis for color change in ΔE^*_{ab}, comparing different bleaching protocols. The analyses are stratified by risk of bias, with "some concerns and low risk of bias" in the upper sections and "high risk of bias" in the lower sections of each comparison. Data are presented as mean difference (IV, Random, 95% CI), with forest plots illustrating the individual and pooled effect sizes. The three comparisons include BGVIOL vs. BG, VIOL vs. BG, and VIOL vs. BGVIOL. Abbreviations: VIOL – violet light only, BGVIOL – bleaching gel combined with violet light, and BG – bleaching gel only.

	BGVIOL			BG				Mean Difference
Study or Subgroup	Mean	SD	Total	Mean	SD	Total	Weight	IV, Random, 95% CI
**2.1.1 Some concerns and low risk of bias**								
Castro 2024	4.8	1.2	22	3.6	1.7	22	17.7%	1.20 [0.33, 2.07]
Kury 2020/2021	14.4	2.2	18	10	4.1	18	11.0%	4.40 [2.25, 6.55]
Martinho 2019	10.3	12.2	31	9.9	8.6	31	3.4%	0.40 [−4.85, 5.65]
Santos 2021	9	4	20	5	2.2	20	11.7%	4.00 [2.00, 6.00]
Zanin 2025	11.5	4.1	28	10.4	3.5	28	11.8%	1.10 [−0.90, 3.10]
**Subtotal (95% CI)**			**119**			**119**	**55.6%**	**2.37 [0.79, 3.95]**
Heterogeneity: Tau² = 2.00; Chi² = 12.82, df = 4 (P = 0.01); I² = 69%								
Test for overall effect: Z = 2.94 (P = 0.003)								
**2.1.2 High risk of bias**								
Costa 2018	12.5	3.6	8	9.6	2.9	12	7.8%	2.90 [−0.09, 5.89]
Lopes 2024	5.1	1.3	20	2.6	0.6	20	18.8%	2.50 [1.87, 3.13]
Rocha 2023	10.3	3.8	16	4.7	0.5	16	12.4%	5.60 [3.72, 7.48]
Trevisan 2022	10	4.5	7	6	2.7	7	5.5%	4.00 [0.11, 7.89]
**Subtotal (95% CI)**			**51**			**55**	**44.4%**	**3.66 [1.84, 5.48]**
Heterogeneity: Tau² = 2.15; Chi² = 9.77, df = 3 (P = 0.02); I² = 69%								
Test for overall effect: Z = 3.94 (P < 0.0001)								
**Total (95% CI)**			**170**			**174**	**100.0%**	**2.92 [1.85, 3.98]**
Heterogeneity: Tau² = 1.47; Chi² = 27.27, df = 8 (P = 0.0006); I² = 71%								
Test for overall effect: Z = 5.37 (P < 0.00001)								
Test for subgroup differences: Chi² = 1.10, df = 1 (P = 0.29), I² = 9.0%								

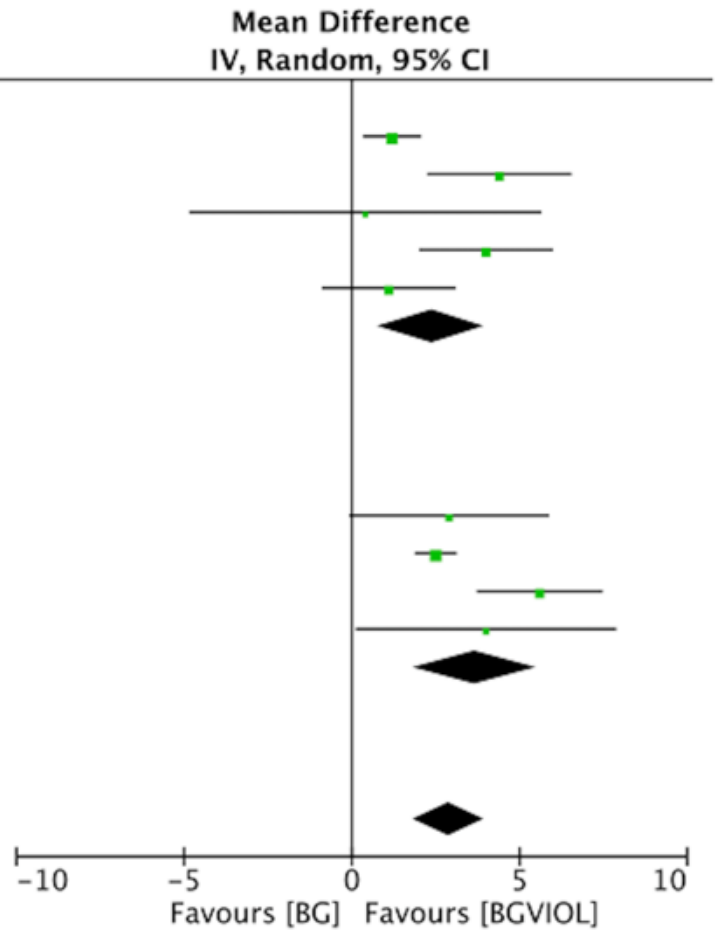

	VIOL			BG				Mean Difference
Study or Subgroup	Mean	SD	Total	Mean	SD	Total	Weight	IV, Random, 95% CI
**1.1.1 Some concerns and low risk of bias**								
Castro 2024	1.2	2.2	22	3.6	17	22	5.1%	−2.40 [−9.56, 4.76]
Kury 2020/2021	3.7	1.4	18	10	4.1	18	14.6%	−6.30 [−8.30, −4.30]
Santos 2021	5	2.2	20	5	2.2	20	15.9%	0.00 [−1.36, 1.36]
Toffol 2017	6.3	4.7	20	10.7	5.6	20	11.7%	−4.40 [−7.60, −1.20]
Zanin 2025	5.1	3	22	10.4	3.5	28	15.0%	−5.30 [−7.10, −3.50]
**Subtotal (95% CI)**			**102**			**108**	**62.4%**	**−3.77 [−6.87, −0.68]**
Heterogeneity: Tau² = 9.98; Chi² = 36.04, df = 4 (P < 0.00001); I² = 89%								
Test for overall effect: Z = 2.39 (P = 0.02)								
**1.1.2 High risk of bias**								
Costa 2018	6.2	3.6	12	9.6	2.9	12	13.1%	−3.40 [−6.02, −0.78]
Panhóca 2022	8	4.7	5	5.2	2.9	5	8.3%	2.80 [−2.04, 7.64]
Rocha 2023	2.2	2.4	16	4.7	0.5	16	16.2%	−2.50 [−3.70, −1.30]
**Subtotal (95% CI)**			**33**			**33**	**37.6%**	**−1.91 [−4.27, 0.44]**
Heterogeneity: Tau² = 2.53; Chi² = 5.02, df = 2 (P = 0.08); I² = 60%								
Test for overall effect: Z = 1.59 (P = 0.11)								
**Total (95% CI)**			**135**			**141**	**100.0%**	**−2.97 [−4.90, −1.05]**
Heterogeneity: Tau² = 5.55; Chi² = 41.87, df = 7 (P < 0.00001); I² = 83%								
Test for overall effect: Z = 3.03 (P = 0.002)								
Test for subgroup differences: Chi² = 0.88, df = 1 (P = 0.35), I² = 0%								

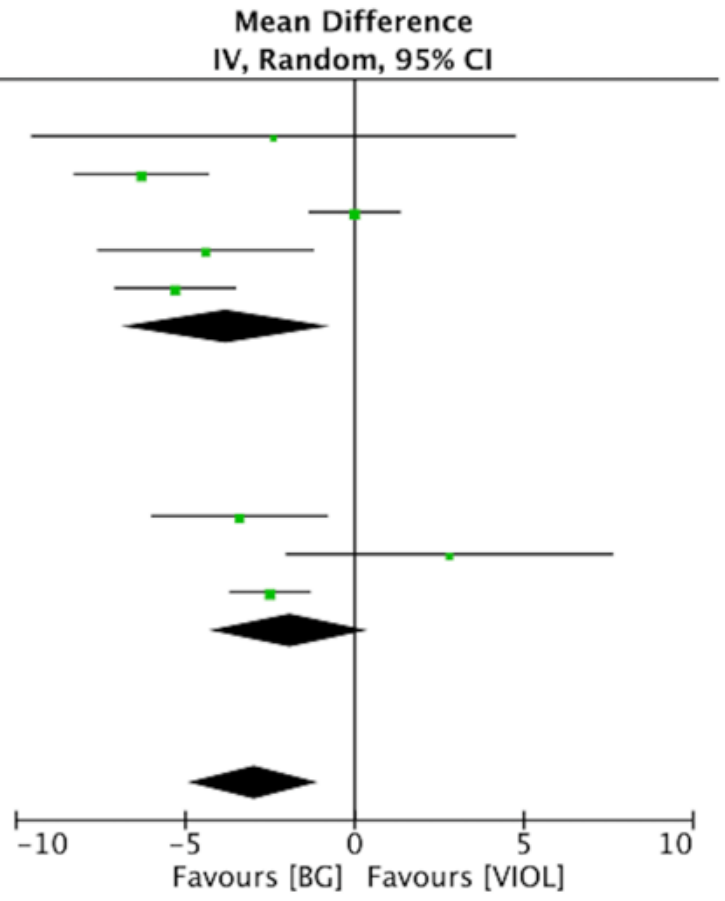

	VIOL			BGVIOL				Mean Difference
Study or Subgroup	Mean	SD	Total	Mean	SD	Total	Weight	IV, Random, 95% CI
**3.1.1 Some concerns and low risk of bias**								
Castro 2024	1.2	2.2	22	4.8	1.2	22	17.8%	−3.60 [−4.65, −2.55]
Kury 2020/2021	3.7	1.4	18	14.4	2.2	18	17.6%	−10.70 [−11.90, −9.50]
Santos 2021	5	2.2	20	9	4	20	16.7%	−4.00 [−6.00, −2.00]
Zanin 2025	5.1	3	22	11.5	4.1	28	16.7%	−6.40 [−8.37, −4.43]
**Subtotal (95% CI)**			**82**			**88**	**68.8%**	**−6.19 [−9.99, −2.39]**
Heterogeneity: Tau² = 14.38; Chi² = 81.87, df = 3 (P < 0.00001); I² = 96%								
Test for overall effect: Z = 3.19 (P = 0.001)								
**3.1.2 High risk of bias**								
Costa 2018	6.2	3.6	12	12.5	3.6	8	14.7%	−6.30 [−9.52, −3.08]
Rocha 2023	2.2	2.4	16	10.3	3.8	16	16.4%	−8.10 [−10.30, −5.90]
**Subtotal (95% CI)**			**28**			**24**	**31.2%**	**−7.53 [−9.34, −5.71]**
Heterogeneity: Tau² = 0.00; Chi² = 0.82, df = 1 (P = 0.37); I² = 0%								
Test for overall effect: Z = 8.11 (P < 0.00001)								
**Total (95% CI)**			**110**			**112**	**100.0%**	**−6.52 [−9.36, −3.69]**
Heterogeneity: Tau² = 11.49; Chi² = 84.19, df = 5 (P < 0.00001); I² = 94%								
Test for overall effect: Z = 4.51 (P < 0.00001)								
Test for subgroup differences: Chi² = 0.39, df = 1 (P = 0.53), I² = 0%								

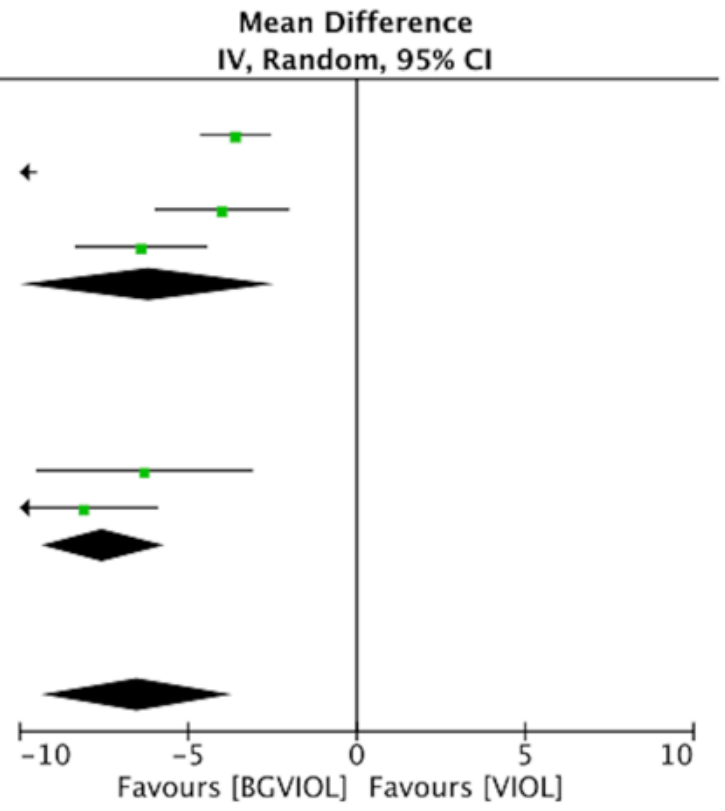

Supplementary Figure 2. Pairwise meta-analysis for **color change in ΔSGU/final SGU**, comparing different bleaching protocols. The analyses are stratified by risk of bias, with "some concerns and low risk of bias" in the upper sections and "high risk of bias" in the lower sections of each comparison. Data are presented as mean difference (IV, Random, 95% CI), with forest plots illustrating the individual and pooled effect sizes. The three comparisons include BGVIOL vs. BG, VIOL vs. BG, and VIOL vs. BGVIOL. *Abbreviations:* VIOL – violet light only, BGVIOL – bleaching gel combined with violet light, and BG – bleaching gel only.

	BGVIOL			BG				Mean Difference
**Study or Subgroup**	**Mean**	**SD**	**Total**	**Mean**	**SD**	**Total**	**Weight**	**IV, Random, 95% CI**
**5.1.1 Some concerns and low risk of bias**								
Castro 2024	2.4	1.6	22	2.4	1.4	22	13.7%	0.00 [−0.89, 0.89]
Kury 2020/2021	6.2	2.1	18	8.7	2	18	12.9%	−2.50 [−3.84, −1.16]
Martinho 2019	5.3	2.3	31	5.1	2.4	31	13.2%	0.20 [−0.97, 1.37]
Zanin 2025	7	2.9	24	7.7	2.5	24	12.5%	−0.70 [−2.23, 0.83]
**Subtotal (95% CI)**			**95**			**95**	**52.4%**	**−0.69 [−1.86, 0.47]**
Heterogeneity: $Tau^2 = 1.02$; $Chi^2 = 11.21$, df = 3 (P = 0.01); $I^2 = 73\%$								
Test for overall effect: Z = 1.17 (P = 0.24)								
**5.1.2 High risk of bias**								
Bugnera 2019	6.1	3.4	25	1.2	1.6	25	12.6%	4.90 [3.43, 6.37]
Costa 2018	6.8	2.2	8	5.4	1.8	12	11.8%	1.40 [−0.43, 3.23]
Lopes 2021	2.3	3.5	35	1	3.3	43	12.5%	1.30 [−0.22, 2.82]
Mayer-Santos 2022	7.1	5.2	25	4.3	3.1	25	10.6%	2.80 [0.43, 5.17]
**Subtotal (95% CI)**			**93**			**105**	**47.6%**	**2.62 [0.74, 4.50]**
Heterogeneity: $Tau^2 = 2.83$; $Chi^2 = 13.75$, df = 3 (P = 0.003); $I^2 = 78\%$								
Test for overall effect: Z = 2.73 (P = 0.006)								
**Total (95% CI)**			**188**			**200**	**100.0%**	**0.86 [−0.63, 2.35]**
Heterogeneity: $Tau^2 = 4.02$; $Chi^2 = 62.96$, df = 7 (P < 0.00001); $I^2 = 89\%$								
Test for overall effect: Z = 1.13 (P = 0.26)								
Test for subgroup differences: $Chi^2 = 8.63$, df = 1 (P = 0.003), $I^2 = 88.4\%$								

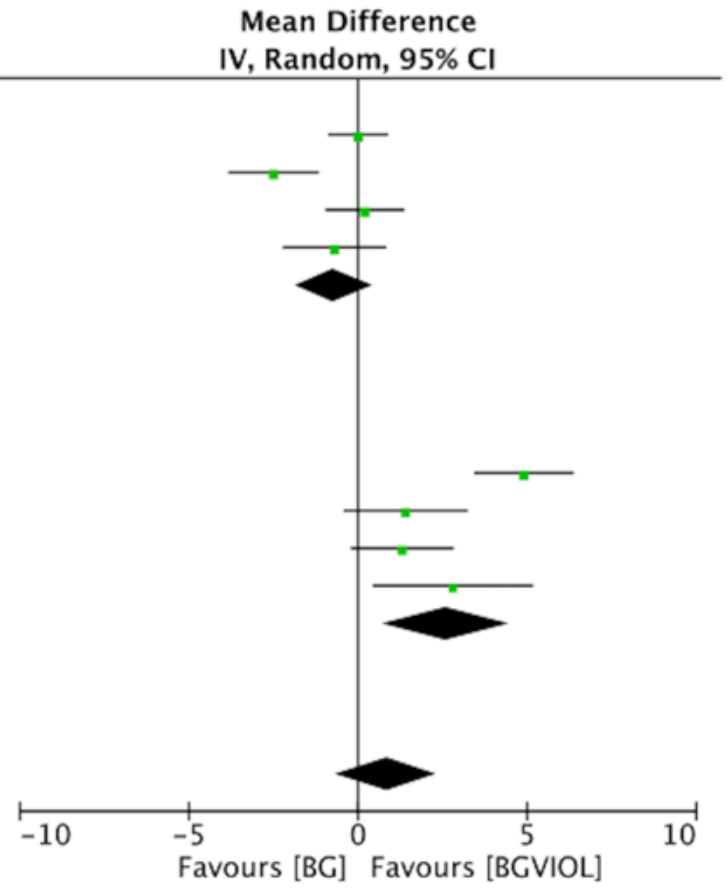

	VIOL			BG				Mean Difference
**Study or Subgroup**	**Mean**	**SD**	**Total**	**Mean**	**SD**	**Total**	**Weight**	**IV, Random, 95% CI**
**4.1.1 Some concerns and low risk of bias**								
Castro 2024	0.5	0.8	22	2.4	1.4	22	15.4%	−1.90 [−2.57, −1.23]
Kury 2020/2021	2.1	2.4	18	8.7	2	18	14.1%	−6.60 [−8.04, −5.16]
Toffol 2017	0.6	2.2	20	6	2.8	20	13.9%	−5.40 [−6.96, −3.84]
Zanin 2025	1.7	2.5	22	7.7	2.5	28	14.2%	−6.00 [−7.40, −4.60]
**Subtotal (95% CI)**			**82**			**88**	**57.7%**	**−4.93 [−7.57, −2.29]**
Heterogeneity: $Tau^2 = 6.82$; $Chi^2 = 57.40$, df = 3 (P < 0.00001); $I^2 = 95\%$								
Test for overall effect: Z = 3.66 (P = 0.0003)								
**4.1.2 High risk of bias**								
Costa 2018	1.6	2.9	12	5.4	1.8	12	13.1%	−3.80 [−5.73, −1.87]
Mayer-Santos 2022	3.9	2	50	4.3	3.1	25	14.4%	−0.40 [−1.74, 0.94]
Sobral 2020	9.7	2	30	11.7	2	30	14.9%	−2.00 [−3.01, −0.99]
**Subtotal (95% CI)**			**92**			**67**	**42.3%**	**−1.95 [−3.59, −0.30]**
Heterogeneity: $Tau^2 = 1.59$; $Chi^2 = 8.48$, df = 2 (P = 0.01); $I^2 = 76\%$								
Test for overall effect: Z = 2.32 (P = 0.02)								
**Total (95% CI)**			**174**			**155**	**100.0%**	**−3.68 [−5.36, −2.00]**
Heterogeneity: $Tau^2 = 4.64$; $Chi^2 = 79.82$, df = 6 (P < 0.00001); $I^2 = 92\%$								
Test for overall effect: Z = 4.30 (P < 0.0001)								
Test for subgroup differences: $Chi^2 = 3.53$, df = 1 (P = 0.06), $I^2 = 71.7\%$								

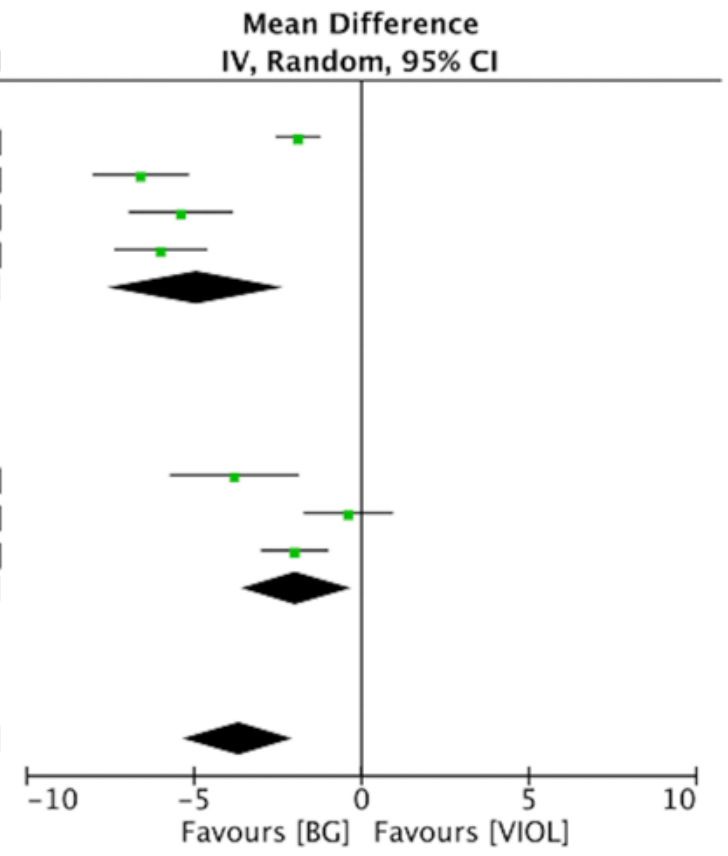

	VIOL			BGVIOL				Mean Difference
**Study or Subgroup**	**Mean**	**SD**	**Total**	**Mean**	**SD**	**Total**	**Weight**	**IV, Random, 95% CI**
**6.1.1 Some concerns and low risk of bias**								
Castro 2024	0.5	0.8	22	2.4	1.6	22	24.0%	−1.90 [−2.65, −1.15]
Kury 2020/2021	2.1	2.4	18	6.2	2.1	18	20.8%	−4.10 [−5.57, −2.63]
Zanin 2025	1.7	2.5	22	7	2.9	28	20.7%	−5.30 [−6.80, −3.80]
**Subtotal (95% CI)**			**62**			**68**	**65.6%**	**−3.69 [−5.88, −1.51]**
Heterogeneity: $Tau^2 = 3.31$; $Chi^2 = 19.24$, df = 2 (P < 0.0001); $I^2 = 90\%$								
Test for overall effect: Z = 3.31 (P = 0.0009)								
**6.1.2 High risk of bias**								
Costa 2018	1.6	2.9	12	6.8	2.2	8	16.9%	−5.20 [−7.44, −2.96]
Mayer-Santos 2022	3.9	2	50	7.1	5.2	25	17.5%	−3.20 [−5.31, −1.09]
**Subtotal (95% CI)**			**62**			**33**	**34.4%**	**−4.16 [−6.12, −2.21]**
Heterogeneity: $Tau^2 = 0.77$; $Chi^2 = 1.62$, df = 1 (P = 0.20); $I^2 = 38\%$								
Test for overall effect: Z = 4.17 (P < 0.0001)								
**Total (95% CI)**			**124**			**101**	**100.0%**	**−3.85 [−5.44, −2.26]**
Heterogeneity: $Tau^2 = 2.59$; $Chi^2 = 23.25$, df = 4 (P = 0.0001); $I^2 = 83\%$								
Test for overall effect: Z = 4.74 (P < 0.00001)								
Test for subgroup differences: $Chi^2 = 0.10$, df = 1 (P = 0.75), $I^2 = 0\%$								

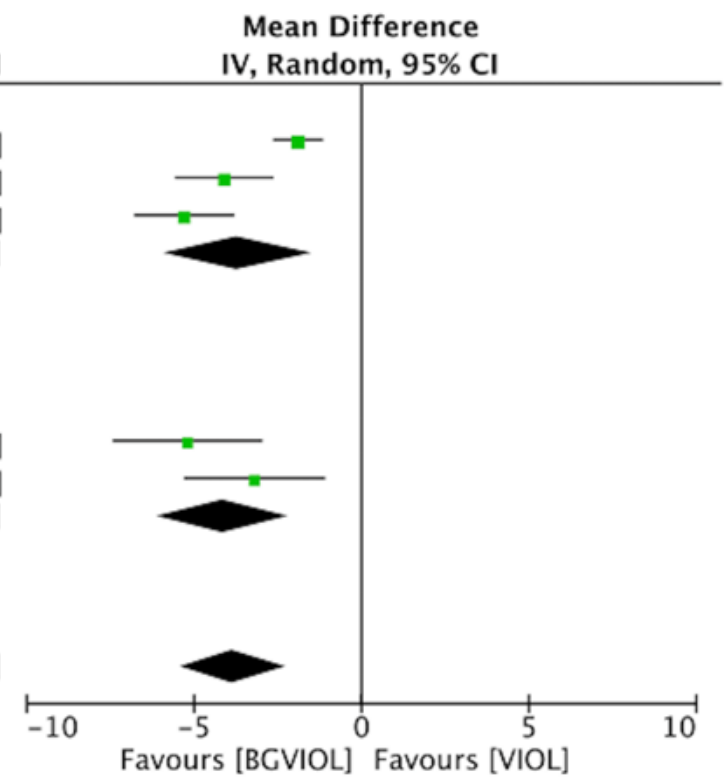

Supplementary Figure 3. Pairwise meta-analysis for risk of TS, comparing different bleaching protocols. The analyses are stratified by risk of bias, with "some concerns and low risk of bias" in the upper sections and "high risk of bias" in the lower sections of each comparison. Data are presented as risk ratio (M-H, Random, 95% CI), with forest plots illustrating individual and pooled effect sizes. The three comparisons include BGVIOL vs. BG, VIOL vs. BG, and VIOL vs. BGVIOL. Abbreviations: VIOL – violet light only, BGVIOL – bleaching gel combined with violet light, and BG – bleaching gel only.

	BGVIOL		BG			Risk Ratio
Study or Subgroup	Events	Total	Events	Total	Weight	M-H, Random, 95% CI
**11.1.1 Some concerns and low risk of bias**						
Castro 2024	8	22	14	22	8.9%	0.57 [0.30, 1.08]
Kury 2020/2021	17	18	16	18	16.7%	1.06 [0.87, 1.30]
Martinho 2019	13	31	13	31	9.7%	1.00 [0.56, 1.80]
Zanin 2025	22	28	12	28	11.6%	1.83 [1.15, 2.93]
**Subtotal (95% CI)**		**99**		**99**	**46.9%**	**1.07 [0.73, 1.56]**
Total events	60		55			
Heterogeneity: $Tau^2 = 0.09$; $Chi^2 = 8.81$, df = 3 (P = 0.03); $I^2 = 66\%$						
Test for overall effect: Z = 0.34 (P = 0.73)						
**11.1.2 High risk of bias**						
Bugnera 2019	2	25	0	25	0.7%	5.00 [0.25, 99.16]
Costa 2018	5	9	13	13	9.9%	0.57 [0.32, 1.01]
Ferreira and Catão 2023	5	9	3	10	4.3%	1.85 [0.61, 5.63]
Lopes 2021	17	35	18	43	11.3%	1.16 [0.71, 1.89]
Lopes 2024	8	20	6	20	6.2%	1.33 [0.57, 3.14]
Mayer-Santos 2022	17	25	25	25	15.4%	0.69 [0.52, 0.90]
Rocha 2023	8	16	4	16	5.2%	2.00 [0.75, 5.33]
**Subtotal (95% CI)**		**139**		**152**	**53.1%**	**1.04 [0.68, 1.60]**
Total events	62		69			
Heterogeneity: $Tau^2 = 0.17$; $Chi^2 = 16.12$, df = 6 (P = 0.01); $I^2 = 63\%$						
Test for overall effect: Z = 0.18 (P = 0.86)						
**Total (95% CI)**		**238**		**251**	**100.0%**	**1.03 [0.79, 1.34]**
Total events	122		124			
Heterogeneity: $Tau^2 = 0.10$; $Chi^2 = 26.62$, df = 10 (P = 0.003); $I^2 = 62\%$						
Test for overall effect: Z = 0.20 (P = 0.84)						
Test for subgroup differences: $Chi^2 = 0.01$, df = 1 (P = 0.92), $I^2 = 0\%$						

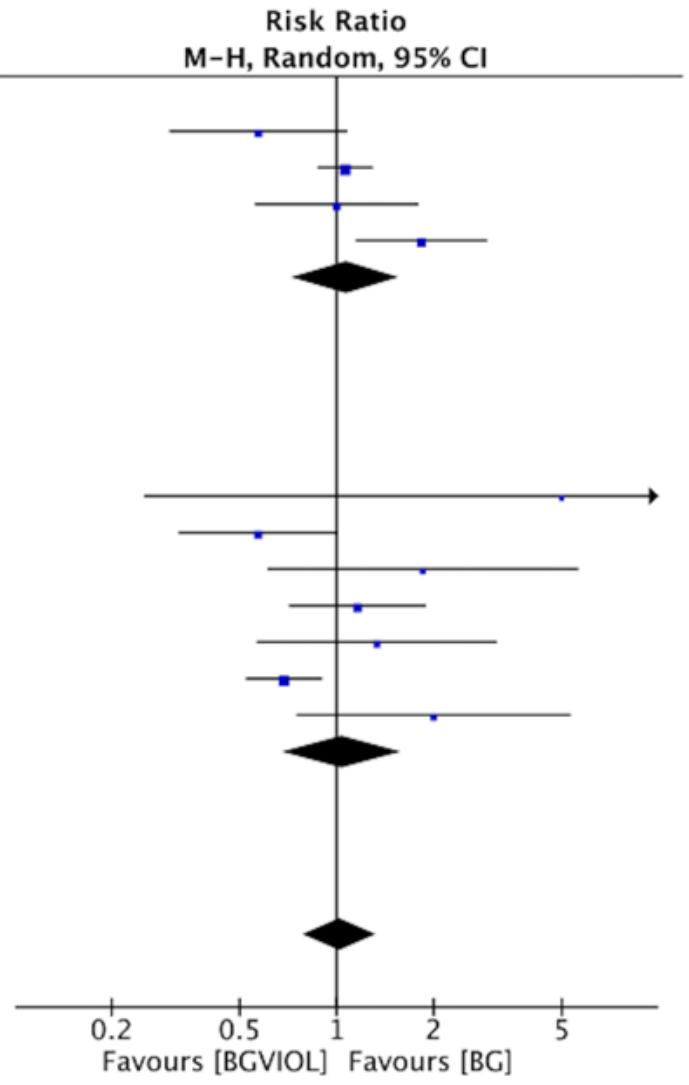

	VIOL		BG			Risk Ratio
Study or Subgroup	Events	Total	Events	Total	Weight	M-H, Random, 95% CI
**10.1.1 Some concerns and low risk of bias**						
Castro 2024	6	22	14	22	15.4%	0.43 [0.20, 0.91]
Kury 2020/2021	1	18	16	18	10.1%	0.06 [0.01, 0.42]
Toffol 2017	3	20	13	20	14.0%	0.23 [0.08, 0.69]
Zanin 2025	16	22	12	28	16.3%	1.70 [1.03, 2.79]
**Subtotal (95% CI)**		**82**		**88**	**55.8%**	**0.38 [0.09, 1.51]**
Total events	26		55			
Heterogeneity: $Tau^2 = 1.67$; $Chi^2 = 28.67$, df = 3 (P < 0.00001); $I^2 = 90\%$						
Test for overall effect: Z = 1.38 (P = 0.17)						
**10.1.2 High risk of bias**						
Costa 2018	3	12	13	13	14.8%	0.28 [0.11, 0.69]
Ferreira and Catão 2023	0	13	3	10	6.8%	0.11 [0.01, 1.95]
Mayer-Santos 2022	7	50	25	25	15.8%	0.15 [0.08, 0.29]
Rocha 2023	0	16	4	16	6.8%	0.11 [0.01, 1.91]
**Subtotal (95% CI)**		**91**		**64**	**44.2%**	**0.18 [0.11, 0.30]**
Total events	10		45			
Heterogeneity: $Tau^2 = 0.00$; $Chi^2 = 1.43$, df = 3 (P = 0.70); $I^2 = 0\%$						
Test for overall effect: Z = 6.50 (P < 0.00001)						
**Total (95% CI)**		**173**		**152**	**100.0%**	**0.27 [0.10, 0.70]**
Total events	36		100			
Heterogeneity: $Tau^2 = 1.39$; $Chi^2 = 50.61$, df = 7 (P < 0.00001); $I^2 = 86\%$						
Test for overall effect: Z = 2.70 (P = 0.007)						
Test for subgroup differences: $Chi^2 = 0.96$, df = 1 (P = 0.33), $I^2 = 0\%$						

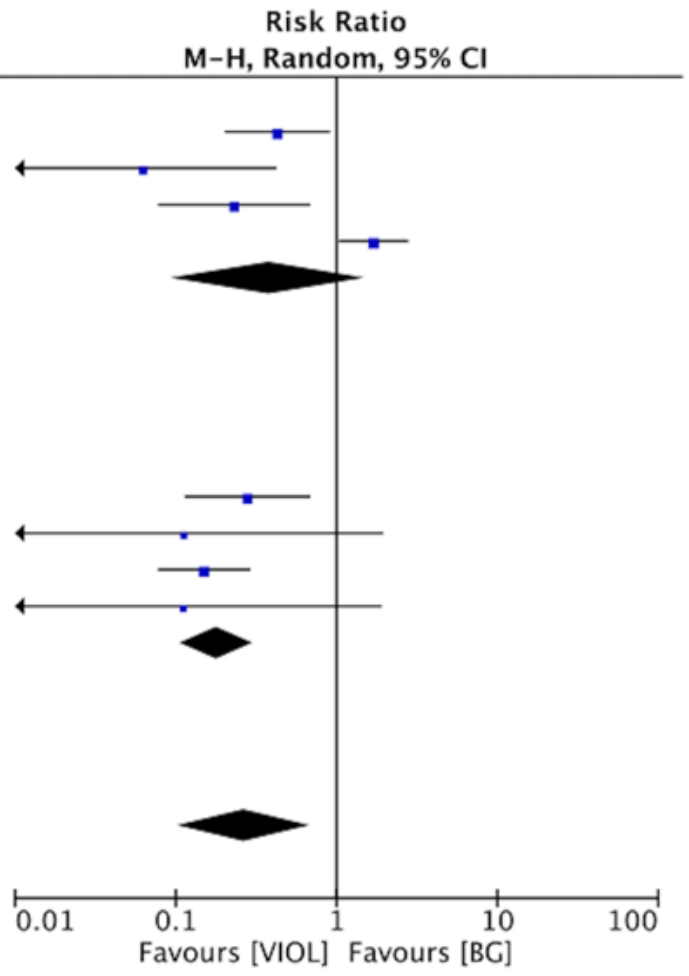

	VIOL		BGVIOL			Risk Ratio
Study or Subgroup	Events	Total	Events	Total	Weight	M-H, Random, 95% CI
**12.1.1 Some concerns and low risk of bias**						
Castro 2024	6	22	8	22	16.7%	0.75 [0.31, 1.80]
Kury 2020/2021	3	18	17	18	15.9%	0.18 [0.06, 0.50]
Zanin 2025	18	22	22	28	18.8%	1.04 [0.79, 1.37]
**Subtotal (95% CI)**		**62**		**68**	**51.4%**	**0.55 [0.16, 1.84]**
Total events	27		47			
Heterogeneity: $Tau^2 = 0.98$; $Chi^2 = 16.91$, df = 2 (P = 0.0002); $I^2 = 88\%$						
Test for overall effect: Z = 0.97 (P = 0.33)						
**12.1.2 High risk of bias**						
Costa 2018	3	12	5	9	15.4%	0.45 [0.14, 1.41]
Ferreira and Catão 2023	0	13	5	9	7.9%	0.06 [0.00, 1.05]
Mayer-Santos 2022	7	50	17	25	17.3%	0.21 [0.10, 0.43]
Rocha 2023	0	16	8	16	7.9%	0.06 [0.00, 0.94]
**Subtotal (95% CI)**		**91**		**59**	**48.6%**	**0.22 [0.11, 0.46]**
Total events	10		35			
Heterogeneity: $Tau^2 = 0.09$; $Chi^2 = 3.54$, df = 3 (P = 0.32); $I^2 = 15\%$						
Test for overall effect: Z = 4.08 (P < 0.0001)						
**Total (95% CI)**		**153**		**127**	**100.0%**	**0.32 [0.11, 0.88]**
Total events	37		82			
Heterogeneity: $Tau^2 = 1.43$; $Chi^2 = 52.14$, df = 6 (P < 0.00001); $I^2 = 88\%$						
Test for overall effect: Z = 2.21 (P = 0.03)						
Test for subgroup differences: $Chi^2 = 1.56$, df = 1 (P = 0.21), $I^2 = 36.0\%$						

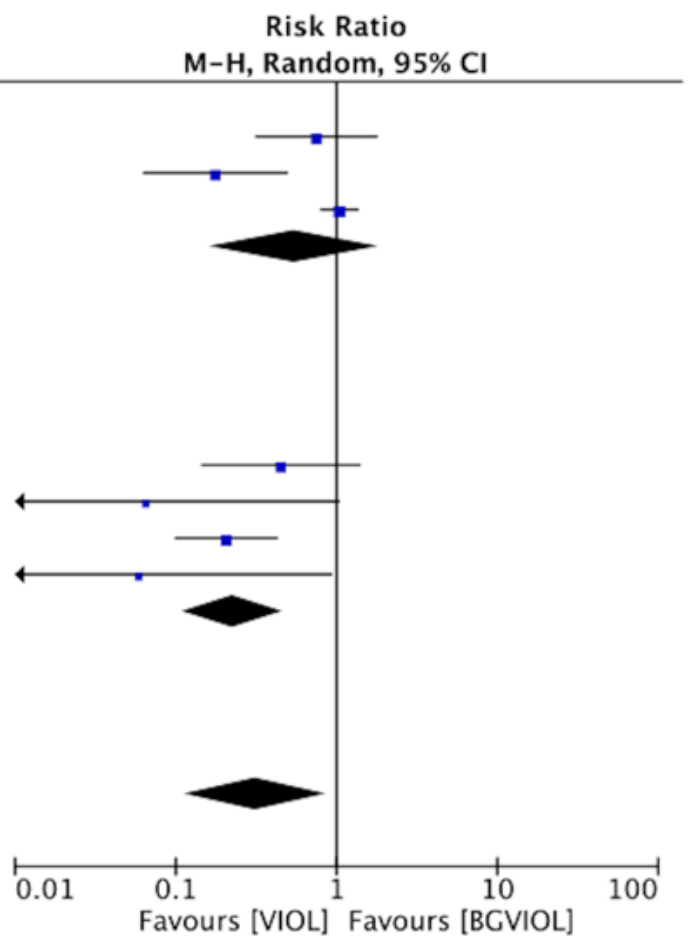

Supplementary Figure 4. Pairwise meta-analysis for intensity of TS, comparing different bleaching protocols. The analyses are stratified by risk of bias, with "some concerns and low risk of bias" in the upper sections and "high risk of bias" in the lower sections of each comparison. Data are presented as mean difference (IV, Random, 95% CI), with forest plots illustrating the individual and pooled effect sizes. The three comparisons include BGVIOL vs. BG, VIOL vs. BG, and VIOL vs. BGVIOL. Abbreviations: VIOL – violet light only, BGVIOL – bleaching gel combined with violet light, and BG – bleaching gel only.

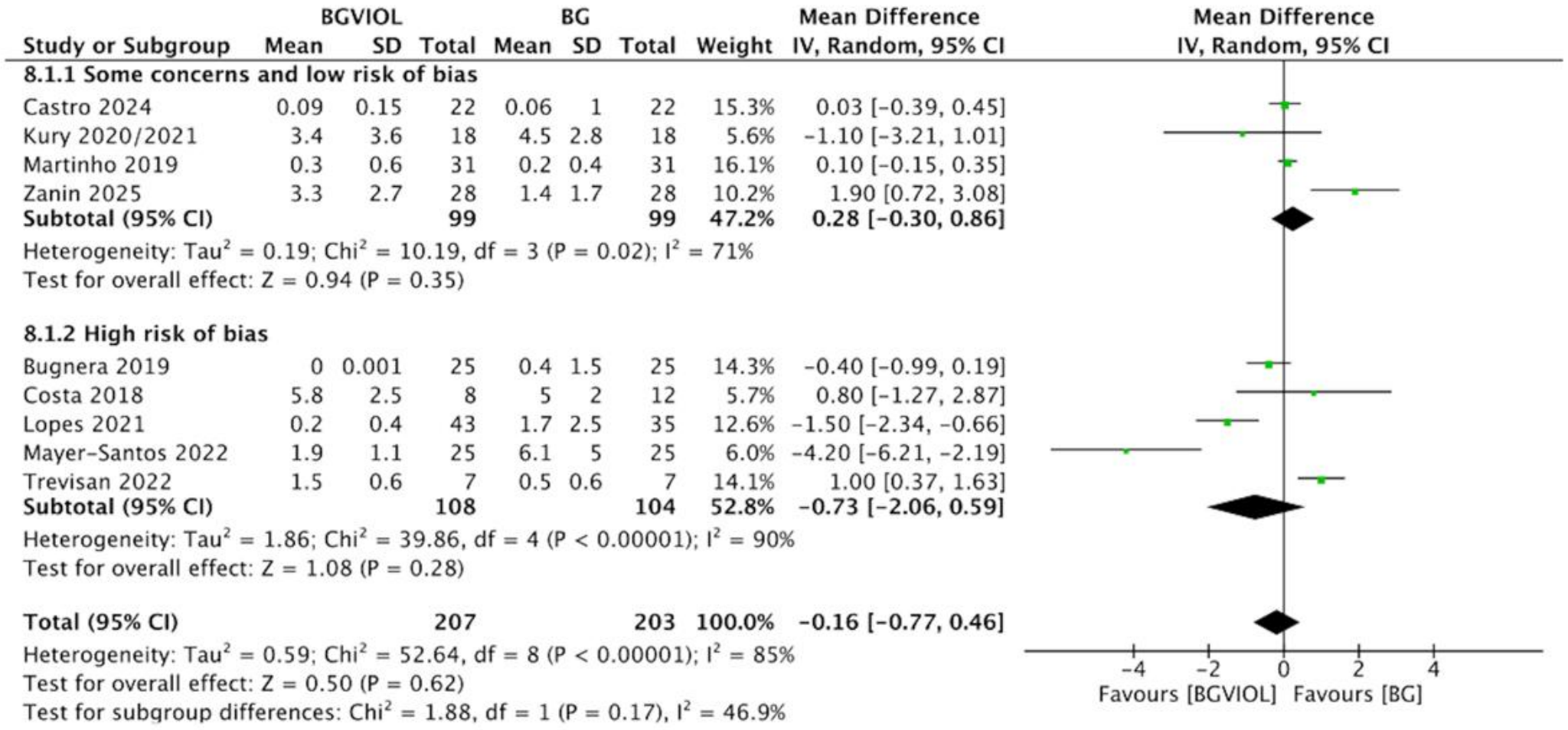

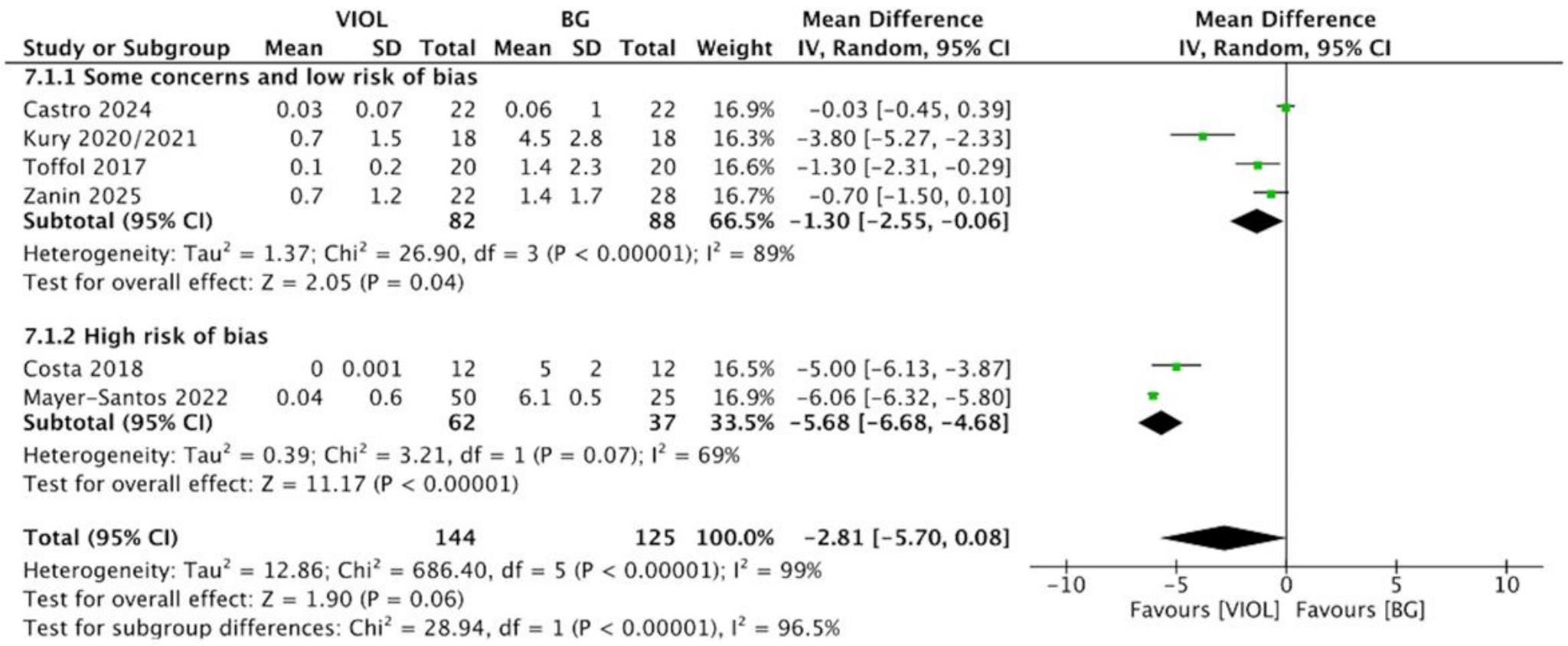

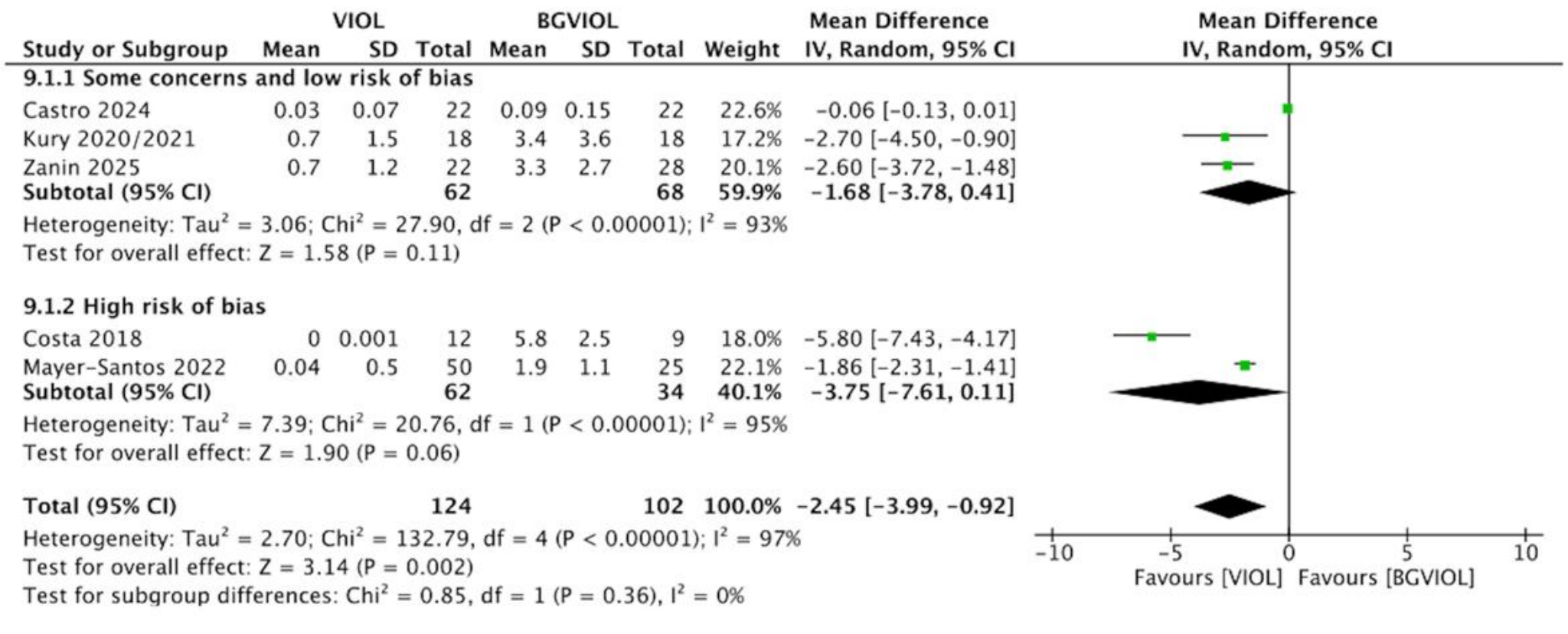

Supplementary Figure 5. Study influence analysis: Leverage versus residual deviation for color change in ΔE^*_{ab}, ΔSGU/final SGU, risk of tooth sensitivity (TS), and intensity of TS. The upper row presents results from all included studies, while the lower row includes only studies with some concerns and low risk of bias. Each plot illustrates the relationship between leverage (influence of a study on the overall model estimates) and residual deviation (degree of misfit between observed and predicted values for each study). Studies positioned further from the origin and near the contour lines indicate higher influence on the model estimates, potentially affecting the robustness of the network meta-analysis results.

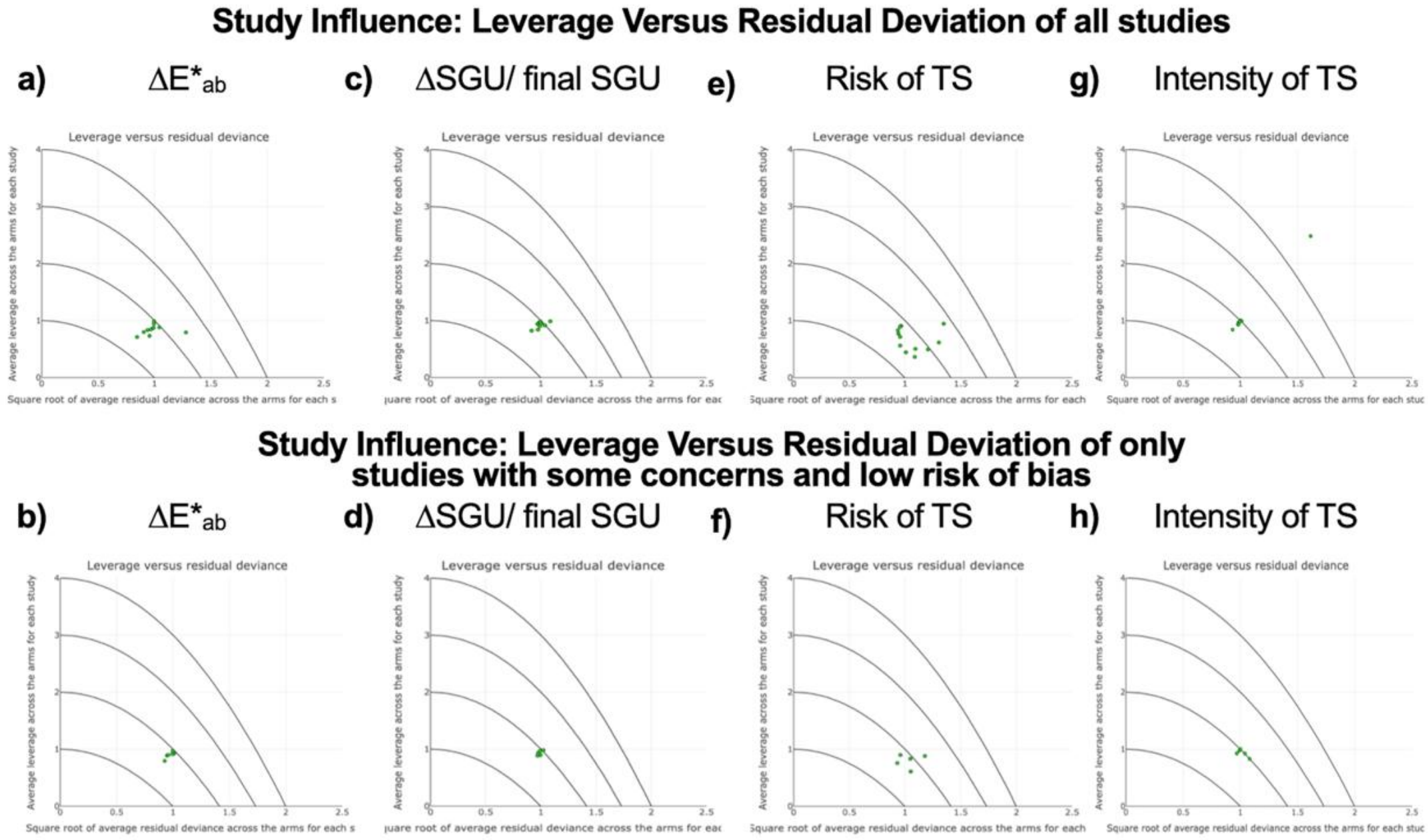

Supplementary Figure 6. Comparison of deviation between the Network Meta-Analysis (NMA) model and the Inconsistency model for color change in ΔE^*_{ab}, ΔSGU/final SGU, risk of tooth sensitivity (TS), and intensity of TS. The upper row presents results from all included studies, while the lower row includes only studies with some concerns and low risk of bias. Each scatter plot compares the deviation from the NMA model (x-axis) with the deviation from the inconsistency model (y-axis), with points aligning along the diagonal line indicating agreement between the two models.

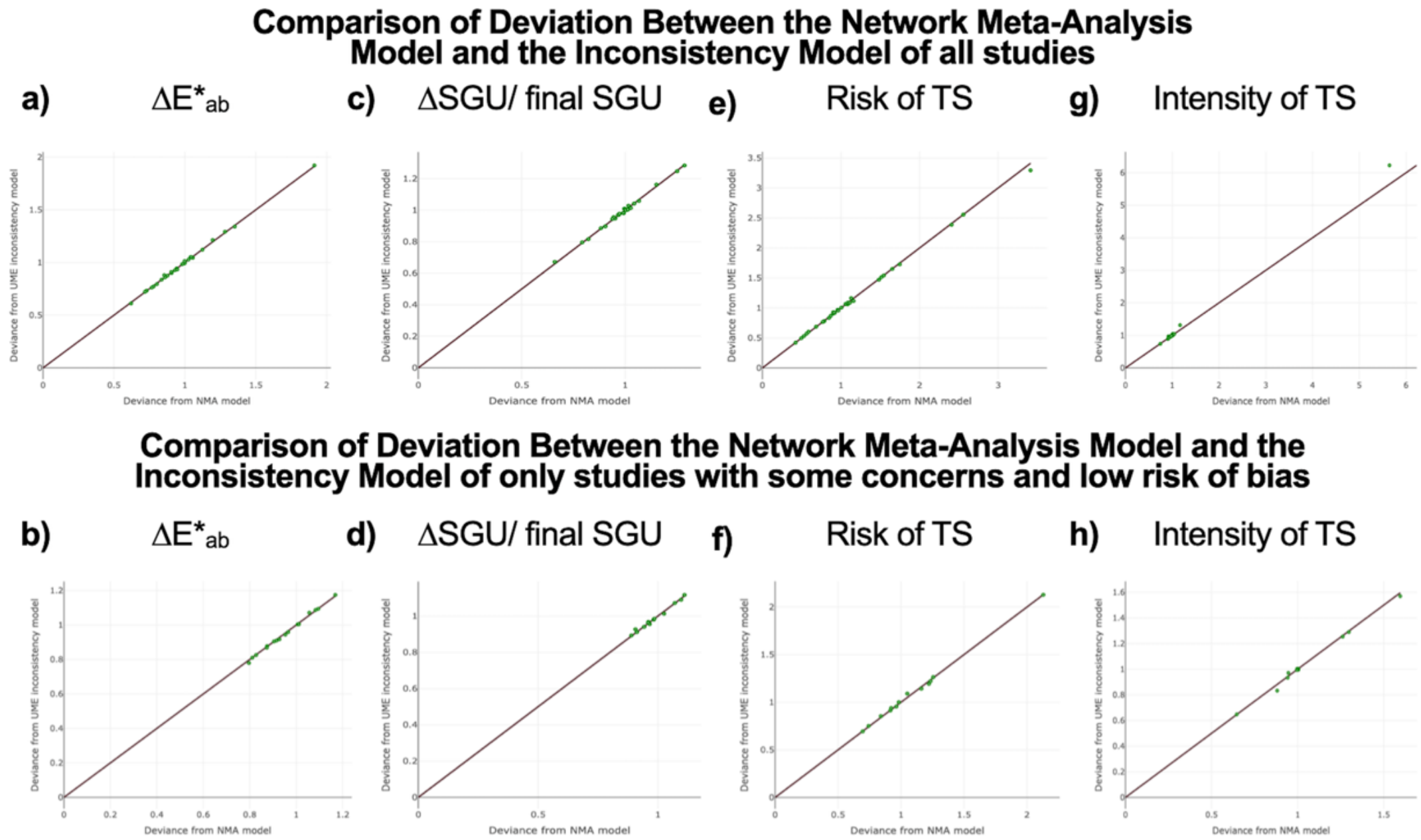

Supplementary Figure 7. Standardized residuals distribution across study arms for color change in ΔE^*_{ab}, ΔSGU/final SGU, risk of tooth sensitivity (TS), and intensity of TS. The upper row presents results from all included studies, while the lower row includes only studies with some concerns and low risk of bias. The residuals represent the differences between observed and predicted values from the network meta-analysis model, providing an assessment of model fit. Higher residuals indicate potential sources of heterogeneity or inconsistency within the included studies.

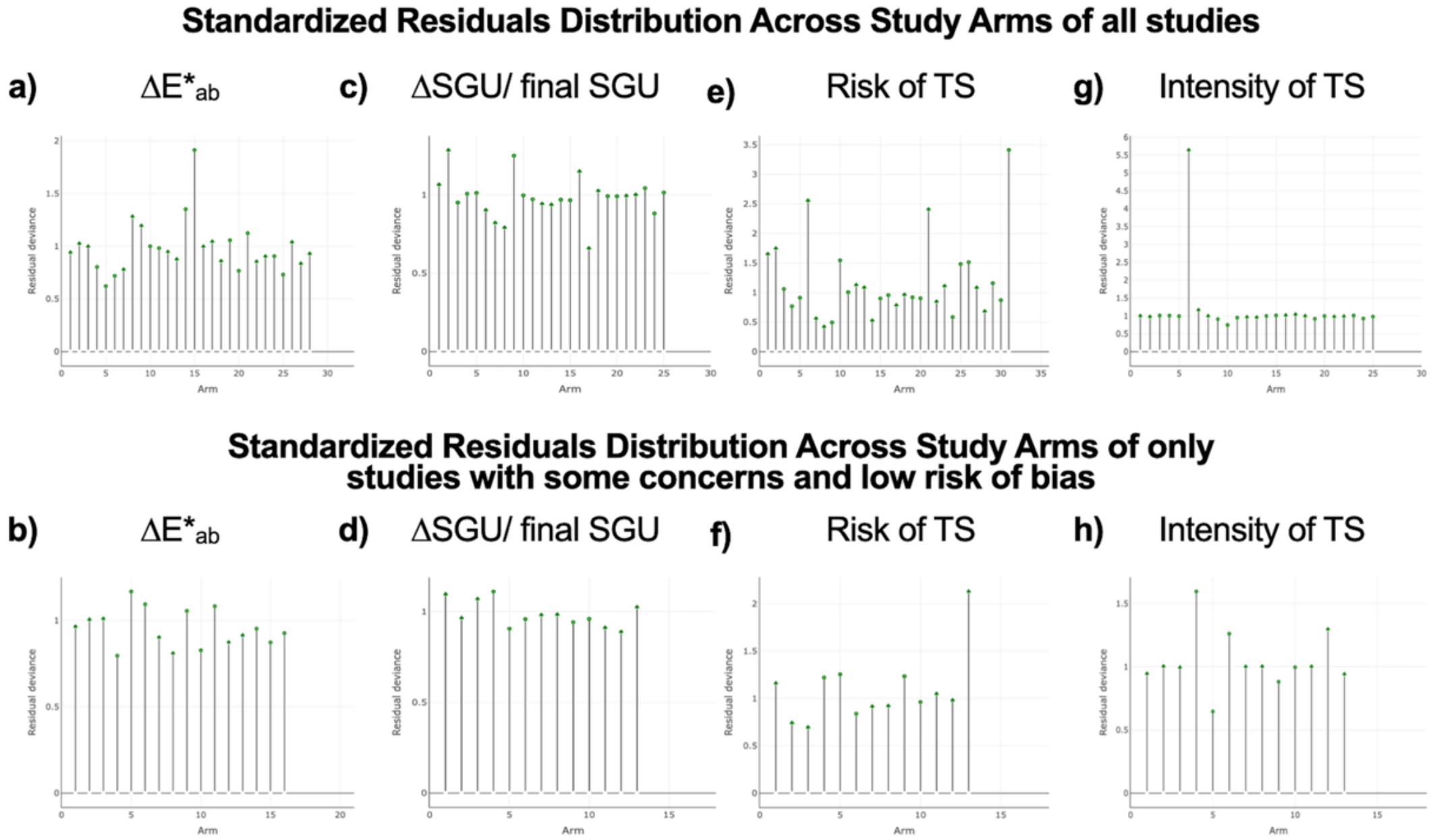

Supplementary Figure 8. Gelman Rubin plot for color change in ΔE^*_{ab}, ΔSGU/final SGU, risk of tooth sensitivity (TS), and intensity of TS. The upper row presents results from all included studies, while the lower row includes only studies with some concerns and low risk of bias.

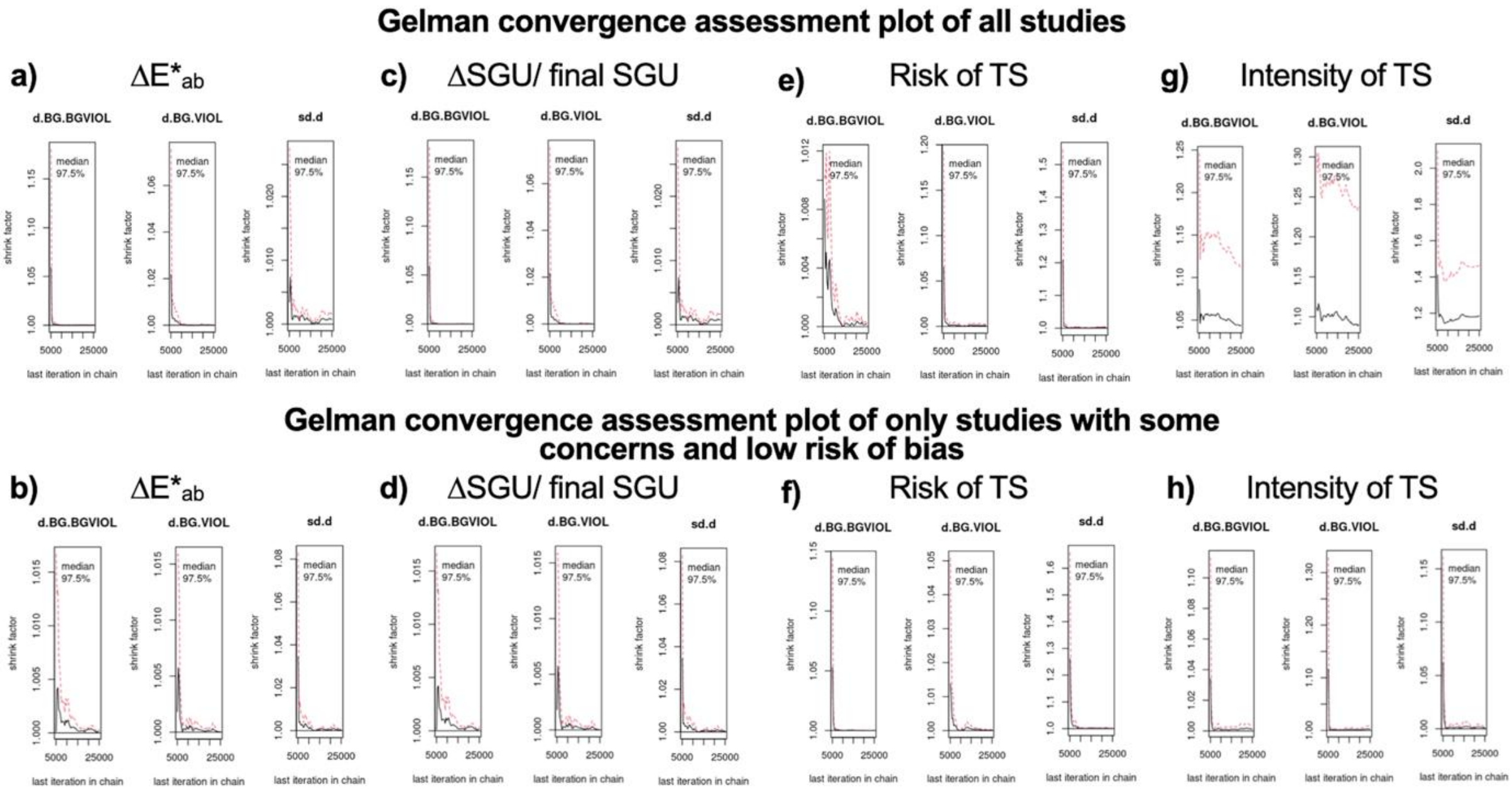

Supplementary Figure 9. Sensitivity analysis excluding studies with imputed data and the study Costa 2018 for intensity of tooth sensitivity (TS). The upper section presents the impact of excluding studies with imputed data on color change in ΔE^*_{ab} and ΔSGU/final SGU, with results expressed as mean difference (95% CrI). The lower section evaluates the effect of removing Costa 2018 on intensity of TS, including a forest plot, a comparison of deviation between the network meta-analysis model and the inconsistency model, a standardized residuals distribution, and a leverage versus residual deviation plot. These analyses assess the robustness of the NMA results when specific data points are excluded.

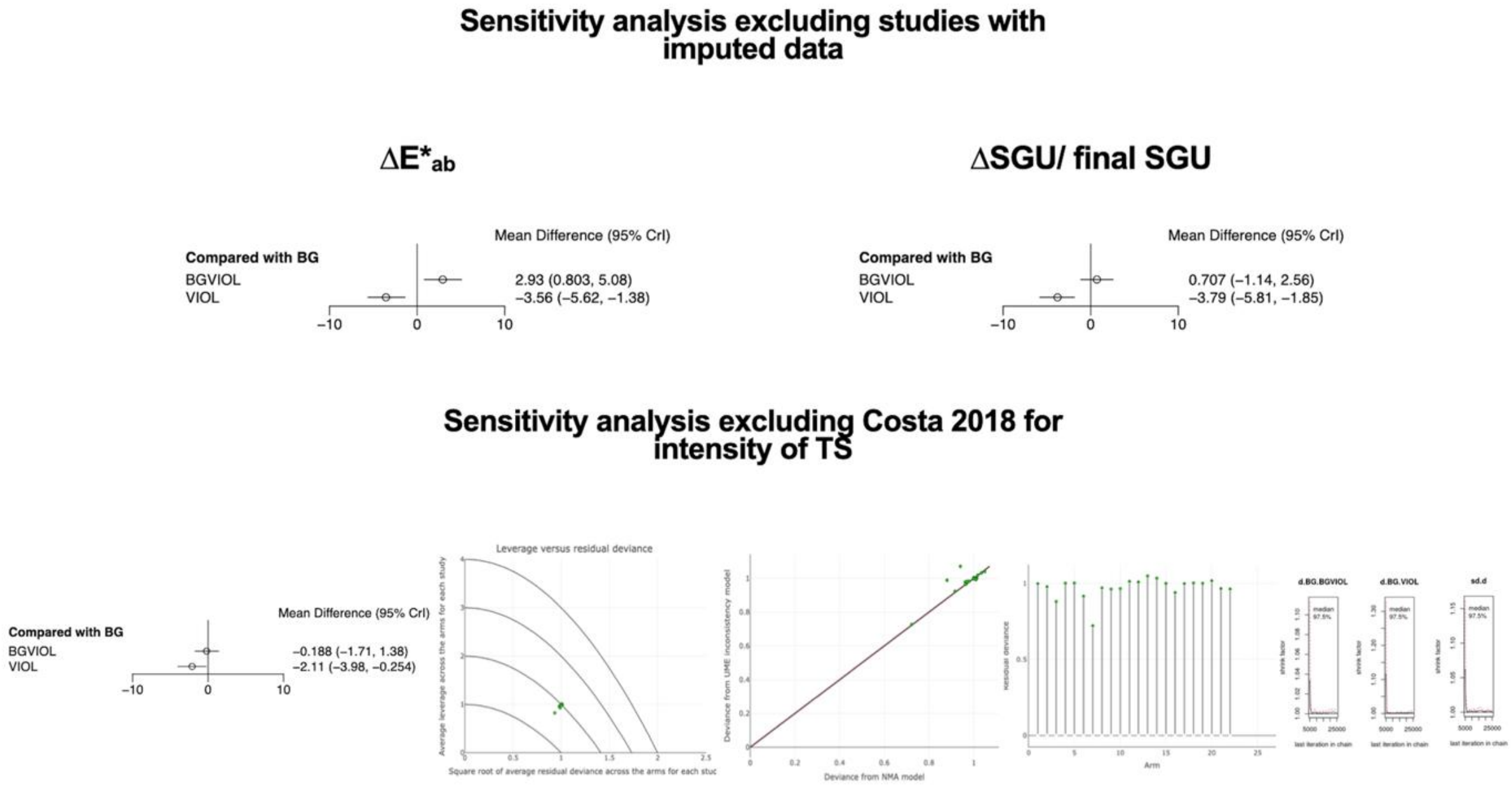

Supplement: Supplementary file 1 [file 1807-3107-bor-40-e056-suppl_fig.pdf]
