# Supplementary material for: Comparative efficacy of violet light, bleaching gel, or their combination, in in-office bleaching: a systematic review and network meta-analysis
Source: Braz Oral Res. 2026 Aug 14;40:e056. doi: 10.1590/1807-3107bor-2026.vol40.056 (PMC13475733; doi:10.1590/1807-3107bor-2026.vol40.056)
Supplement: Supplementary file 2 [file 1807-3107-bor-40-e056-suppl_table.pdf]

Supplementary Table 1. Electronic database and search strategy. Search performed on June 27, 2023 (updated on March 15, 2025).

**PUBMED = 1532**		
#1 "Tooth Discoloration"[MeSH Terms] OR "Tooth Discoloration"[Title/Abstract] OR "tooth staining"[Title/Abstract] OR "teeth staining"[Title/Abstract] OR "tooth stain"[Title/Abstract] OR " stained tooth"[Title/Abstract] OR "stained teeth"[Title/Abstract] OR "teeth discoloration"[Title/Abstract] OR "tooth discoloration"[Title/Abstract] OR "teeth discoloration"[Title/Abstract] OR " discolored tooth"[Title/Abstract] OR "discolored teeth"[Title/Abstract] OR "discolored tooth"[Title/Abstract] OR "discolored teeth"[Title/Abstract] OR "dental discoloration"[Title/Abstract] OR " dental discolouration"[Title/Abstract] OR "tooth color"[Title/Abstract] OR "tooth colour"[Title/Abstract] OR "teeth color"[Title/Abstract] OR "teeth colour"[Title/Abstract]	#2 "tooth bleaching"[MeSH Terms] OR "tooth bleaching agents"[MeSH Terms] OR "hydrogen peroxide"[MeSH Terms] OR "carbamide peroxide"[MeSH Terms] OR "Photobleaching"[MeSH Terms] OR "bleaching"[Title/Abstract] OR "whitening"[Title/Abstract] OR "hydrogen peroxide"[Title/Abstract] OR "carbamide peroxide"[Title/Abstract] OR "in office"[Title/Abstract] OR "light activation"[Title/Abstract] OR "light activated"[Title/Abstract] OR "violet led"[Title/Abstract]	
**#1 AND #2**		
**SCOPUS = 1162**		
#1 TITLE-ABS-KEY ("t??th discoloration" OR "t??th staining" OR "tooth stain" OR "stained t??th" OR "t??th discolouration" OR "discolored t??th" OR "discoloured t??th" OR "dental discolo*" OR "t??th color" OR "t??th colour")	#2 TITLE-ABS-KEY ("t??th bleaching" OR "t??th bleaching agents" OR "hydrogen peroxide" OR "carbamide peroxide" OR "Photobleaching" OR "bleaching" OR "whitening" OR "hydrogen peroxide" OR "carbamide peroxide" OR "in office" OR "light activat*" OR "violet led")	#3 (LIMIT-TO (SUBAREA , "DENT"))
**#1 AND #2**		
**WEB OF SCIENCE = 699**		
#1 TS= ("t??th discoloration" OR "t??th staining" OR "tooth stain" OR "stained t??th" OR "t??th discolouration" OR "discolored t??th" OR "discoloured t??th" OR "dental discolo$" OR "t??th color" OR "t??th colour")	#2 TS= ("t*th bleaching" OR "tooth bleaching agents" OR "hydrogen peroxide" OR "carbamide peroxide" OR "photobleaching" OR bleaching OR whitening OR "in office" OR "light activat*" OR "violet led")	
**#1 AND #2**		
**LILACS AND BBO = 289**		
#1 (MH:(Tooth Discoloration)) OR (MH:(Dentition, Permanent)) OR (tw:("tooth color")) OR (tw:("tooth colour")) OR (tw:("teeth color")) OR (tw:("teeth colour")) OR (tw:("tooth staining")) OR (tw:("tooth stain")) OR (tw:("stained tooth")) OR (tw:("stained teeth")) OR (tw:("tooth discoloration")) OR (tw:("tooth discolouration")) OR (tw:("discolored tooth")) OR (tw:("discoloured tooth")) OR (tw:("discolored teeth")) OR (tw:("discoloured teeth")) OR (tw:("teeth discoloration")) OR (tw:("teeth discolouration")) OR (tw:("dental discoloration")) OR (tw:("dental discolouration")) OR (tw:("tooth discolorations")) OR (tw:("color del diente")) OR (tw:("Color de los dientes")) OR (tw:("Manchas en los dientes")) OR (tw:("Manchas dentales")) OR (tw:("Diente manchado")) OR (tw:("dientes manchados")) OR (tw:("Descoloración de los dientes")) OR (tw:("Dientes descoloridos")) OR (tw:("Dientes de descoloración")) OR (tw:("Descoloración dental")) OR (tw:("descoloraciones de los dientes")) OR (tw:("cor dental")) OR (tw:("Cor dos dentes")) OR (tw:("Dente escurecido")) OR (tw:("Dentes escurecidos")) OR (tw:("Dente manchado")) OR (tw:("Dentes manchados")) OR (tw:("Descoloração dos dentes")) OR (tw:("Descoloração dentária")) OR (tw:("Dente descolorido")) OR (tw:("Descoloração dental")) OR (tw:("descolorações dos Dentes"))	#2 (MH:("Tooth Bleaching")) OR (MH:("Tooth Bleaching Agents")) OR (MH:("Hydrogen Peroxide")) OR (MH:("Carbamide Peroxide")) OR (MH:("Photobleaching")) OR (tw:(Bleaching)) OR (tw:(Clareamiento)) OR (tw:(Blanqueamiento)) OR (tw:("Clareamento Dental")) OR (tw:(Whitening)) OR (tw:("Hydrogen Peroxide")) OR (tw:("Peróxido de Hidrógeno")) OR (tw:("Peróxido de Hidrogênio")) OR (tw:("Carbamide Peroxide")) OR (tw:("Peróxido de Carbamida")) OR (tw:("in office")) OR (tw:("em Consultório")) OR (tw:("en el consultorio")) OR (tw:("light activation")) OR (tw:("activacón de la luz")) OR (tw:("light activated")) OR (tw:("activado por la luz")) OR (tw:("ativo com luz"))OR (tw:("violet led")) OR (tw:("led violeta"))	#3 db: ("LILACS" OR "BBO")
**#1 AND #2**		

COCHRANE LIBRARY = 587			
#1	MeSH descriptor: [Tooth Discoloration] explode all trees	#11	MeSH descriptor: [Tooth Bleaching] explode all trees
#2	MeSH descriptor: [Dentition, Permanent] explode all trees	#12	MeSH descriptor: [Tooth Bleaching Agents] explode all trees
#3	(stain next teeth*):ti,ab,kw (Word variations have been searched)	#13	MeSH descriptor: [hydrogen peroxide] explode all trees
#4	(tooth next stain*):ti,ab,kw (Word variations have been searched)	#14	MeSH descriptor: [carbamide peroxide] explode all trees
#5	(tooth* next discoloration):ti,ab,kw (Word variations have been searched)	#15	MeSH descriptor: [Photobleaching] explode all trees
#6	(discoloration next tooth*):ti,ab,kw (Word variations have been searched)	#16	(bleaching):ti,ab,kw (Word variations have been searched)
#7	(tooth* next color):ti,ab,kw (Word variations have been searched)	#17	(whitening):ti,ab,kw (Word variations have been searched)
#8	(teeth* next discoloration*):ti,ab,kw (Word variations have been searched)	#18	(hydrogen next peroxide):ti,ab,kw (Word variations have been searched)
#9	(color next tooth*):ti,ab,kw (Word variations have been searched)	#19	(carbamide next peroxide):ti,ab,kw (Word variations have been searched)
#10	#1 or #2 or #3 or #4 or #5 or #6 or #7 or #8 or #9	#20	(in office):ti,ab,kw (Word variations have been searched)
		#21	(light next activat*):ti,ab,kw (Word variations have been searched)
		#21	(violet led):ti,ab,kw (Word variations have been searched)
		#23	#11 or #12 or #13 or #14 or #15 or #16 or #17 or #18 or #19 or #20 or #21 or #22
		#24	#10 AND #22

EMBASE = 587		
#1 'tooth discoloration'/exp OR 'tooth discoloration' OR 'tooth staining'/exp OR 'tooth staining' OR 'teeth staining' OR 'tooth stain'/exp OR 'tooth stain' OR 'stained tooth' OR 'stained teeth' OR 'teeth discoloration' OR 'tooth discolouration'/exp OR 'teeth discolouration' OR 'discolored tooth' OR 'discolored teeth' OR 'discoloured tooth' OR 'discoloured teeth' OR 'dental discoloration'/exp OR 'dental discoloration' OR 'dental discolouration'/exp OR 'dental discolouration' OR 'tooth color'/exp OR 'tooth color' OR 'tooth colour'/exp OR 'tooth colour' OR 'teeth color' OR 'teeth colour'	#2 'tooth bleaching'/exp OR 'tooth bleaching' OR 'tooth bleaching agents'/exp OR 'tooth bleaching agents' OR 'photobleaching'/exp OR 'photobleaching' OR 'bleaching'/exp OR 'bleaching' OR 'whitening'OR 'hydrogen peroxide'/exp OR 'hydrogen peroxide' OR 'carbamide peroxide'/exp OR 'carbamide peroxide' OR 'in office' OR 'light activation' OR 'light activated' OR 'violet led'	#3 [embase]/lim
**#1 AND #2 NOT MEDLINE**		
**GOOGLE SCHOLAR = 100**		
tooth bleaching AND violet light		

Supplementary Table 2. Individual characteristics of each study. The characteristics of the groups, protocols, and assessment methods were extracted and described only for the groups of interest, as specified in the 'Data items and outcomes - Decision-Making data items and outcomes' section.

Study ID	Study Design [setting]	Mean Age [Mean ± SD] [range, years]	# subjects [%, male]	Baseline color/ evaluated tooth	Groups & Materials [# per group]	Bleaching Protocol Applications x min [sessions] – interval days	Light Protocol: Applications x time, min (resting time, s) [# sessions] – interval days	Wavelength, nm – Irradiance, mW/cm^2 - Power output, W	Color evaluation tools [color change outcomes]	TS Scale [outcomes]	Follow-up [Drop-outs]
Brugner, 2019	Parallel [University]	27.6 ± 4.7 [20-39]	50 [22]	A2/Canine and incisor	I: CP 35%[a] [25]<br>II: CP 35%[a] + light[b] [25]	I: 1 x 30' [2] - 7<br>II: 1 x 30' [2] - 7	I: no light<br>II: 20 x 1' (30'') [2] - 7	405 to 410 nm -112 mW/cm^2/1.2 W (4 LEDs de 300 mW)	Spectrophotometer[c] [ΔSGU] and shade guide[d] [ΔSGU]	VAS 0-10 [Spontaneous TS risk and intensity]	14 days [0]
Castro, 2024	Parallel [University]	23.5 ± 4.9 [18-n.r.]	66 [62]	A2/Incisor	I: HP 6%[e] [22]<br>II: HP 6%[e] + Light[b] [22]<br>III: Light[b] [22]	I: 1 x 30' [2] - 7<br>II: 1 x 30' [2] - 7<br>III: no BG	I: no light<br>II: 20 x 1' (30'') [2] - 7<br>III: 20 x 1' (30'') [2] - 7	405 to 410 nm -112 mW/cm^2/1.2 W (4 LEDs de 300 mW)	Spectrophotometer[f] [ΔE^*_{ab}] and shade guides[d] [ΔSGU]	VAS 0-10 [Spontaneous TS risk and intensity]	30 days [0]
Costa, 2018	Parallel [University]	22.1 ± 3.2 [18-35]	38 [40]	C2/Incisor	I: HP 35%[g] [13]<br>II: Light[b] + HP35%[g] [13] III: Light[b] [12]	I: 3 x 15' [3] - 7<br>II: 1 x 45' [3] – 7<br>III: no BG	I: no light<br>II: 20 x 1' (30'') [3] – 7<br>III: 20 x 1' (30'') [4] - 7	405 ± 10/n.r. /n.r. (4 LEDs n.r.)	Spectrophotometer[f] [ΔE^*_{ab} and ΔSGU]	VAS 0-100 [Spontaneous TS risk and intensity]	30 days [4]
Ferreira and Catão, 2023	Parallel [University]	27.4 ± 5.1 [18-45]	45 [60]	n.r./Canine & incisor	I: HP 35%[h] [15]<br>II: HP 35%[h] + Light[b] [15]<br>III: Light[b] [15]	I: 3 x 15' [3] - 7<br>II: 3 x 15' [3] - 7<br>III: no BG	I: no light<br>II: 1 x 3' (n.r.) [3] - 7<br>III: 1 x 20' (n.r.) [3] - 7	405 ± 10 nm/140.2 mW/cm^2/1.2	Spectrophotometer[f] [final SGU]	VAS 0-10 [Spontaneous TS risk]	30 days [13]
Kury, 2020/ 2021	Parallel [University]	21.5 ± 4.1 [18-60]	100 [32]	A2/Canine	I: Light[b] [18]<br>II: Light[b] + HP 35%[g] [18]<br>II: HP 35%[g] [18]	I: no BG<br>II: 1 x 30' [3] - 7<br>III: 1 x 30' [3] - 7	I: 20 x 1' (30'') [8] - 4<br>II: 20 x 1' (30'') [3] - 7<br>III: no light	405 nm - 112 mW/cm^2/1.2 W (4 LEDs de 300 mW)	Spectrophotometer[f] [ΔE^*_{ab}] and shade guide[d] [ΔSGU]	VAS 0-10 [Spontaneous TS risk and intensity]	14 days [10]
Lopes, 2024	Parallel [University]	24.9 ± 4.2 [18-35]	40 [n.r.]	A2/n.r.	I: Light[b] [20]<br>II: CP 37%[i] + Light[b] [20]	I: no BG<br>II: 5 x 7'30'' [2] – 7	I: 10 x 2' (30'') [2] - 7<br>II: 3 x 2' (30'') [5] - 7	405 ± 10 nm /425 mW/cm^2/1.7 W	Spectrophotometer[f] [ΔE^*_{ab}]	VAS 0-10 [Spontaneous TS risk and intensity]	30 days [0]

Lopes, 2021	Parallel [University]	n.r. ± n.r. [18-n.r.]	102 [n.r.]	n.r./Incisor	I: HP 35%[j] + Light[k] [51]<br>II: HP 35%[j] [51]	I: n.r. x 18' [n.r.] - n.r.<br>II: 1 x 45' [n.r.] - n.r.	I: 1 x 18' (n.r.) [n.r.] - n.r.<br>II: no light	405 – 410 nm /n.r./n.r.	Spectrophotometer[f] [final SGU]	VAS 0-10 [Spontaneous and Stimuli-induced TS risk and intensity]	7 days [41]
Martinho, 2019	Split-Mouth [University]	22.5 ± 3.2 [18-40]	31 [n.r.]	C2/Canine	I: HP 6%[e] + Light[b] [31]<br>II: HP 6%[e] [31]	I: 1 x 33' [2] - 7<br>II: 1 x 33' [2] - 7	I: 20 x 1' (30") [2] - 7<br>II: no light	405-410 nm/n.r./n.r.	Spectrophotometer[f] [ΔE^*_{ab}] and shade guides[d] [ΔSGU].	VAS 0-10 [Spontaneous TS risk and intensity]	30 days [0]
Mayer-Santos, 2022	Parallel [University]	22.3 ± 2.1 [18-30]	100 [n.r.]	A3/Incisor & Canine	I: HP 35%[a] [25]<br>II: Light[b] [25]<br>III: Light[b] [25]<br>IV: Light[b] + HP35%[a] [25]	I: 1 x 45' [4] - 7<br>II: no BG<br>III: no BG<br>IV: 1 x 15' [4] - 7	I: no light<br>II: 20 x 1' (30") [4] - 7<br>III: 20 x 1' (30") [4] - 3<br>IV: 20 x 1' (30") [4] - 7	405 ± 10 nm/112 mW/cm^2/1.2 W	Spectrophotometer[l] [ΔE^*_{ab}] and shade guide[d] [ΔSGU]	VAS 0-100 [Spontaneous TS risk and Intensity]	14 days [0]
Panhóca, 2022	Parallel [University]	n.r. ± n.r. [18.-40	13 [n.r.]	n.r./Incisor, Canine, Lateral incisors, first pre-molares	I: Light[b] [5]<br>II: HP 35%[m] + Light[b] [5]	I: no BG<br>II: n.r. x n.r. [2] - n.r.	I: 20 x 30" (1') [2] – n.r.<br>II: 20 x 30" (1') [2] – n.r.	408 nm /165 mW/cm^2/1400 mW (4 LEDs de 350 mW)	Shade guide[d] [n.r.] and colorimeter[n] [ΔE^*_{ab}]	VAS 0-10 [Provoked TS risk]	Initial [0]; before [0]; after each session [3].
Rocha, 2023	Parallel [University]	n.r. ± n.r. [18-40]	16 [n.r]	A2/incisor & canine	I: Placebo[o] + Light[b] [16]<br>II: HP 35%[g] [16]<br>III: HP 35%[g] + Light[b] [16]	I: no BG<br>II: 2 x 15' [2] - 7<br>III: 2 x 15' [2] - 7	I: 10 x 1' (30") [2] - 7<br>II: no light<br>III: 10 x 1' (30") [2] - 7	405 to 410 nm/n.r./n.r.	Spectrophotometer[f] [ΔE^*_{ab}] and shade guide[d] [ΔSGU]	VAS 0-100 [Spontaneous TS risk]	30 days [0]
Santos, 2021	Parallel [University]	24.5 ± 4.5 [18-40]	80 [31]	A2/Canine	I: Light[b] [20]<br>II: Light[b] + CP35%[p] [20]<br>III: CP 35%[p] [20]	I: no BG<br>II: 1 x 30' [4] - 7<br>III: 1 x 30' [4] - 7	I: 20 x 1' (30") [4] - 7<br>II: 20 x 1' (30") [4] - 7<br>III: no light	400 ± 10 nm/112 mW/cm^2/1.2 W	Spectrophotometer[l] [ΔE^*_{ab}]	VAS 0-10 [n.r.]	15 days [2]; 180 days [5]
Sobral, 2020	Parallel [University]	n.r. ± n.r. [18-30]	60 [n.r.]	A2/Incisor & canine	I: 35% HP[m] [30]<br>II: Light[b] [30]	I: 1 x 40' [2] - 7<br>II: no BG	I: no light<br>II: 20 x 1' (30") [2] - 7	405 to 410 nm /112 mW/cm^2/1.2 W (4 LEDs de 350 mW)	Shade guide[d] [final SGU]	VAS 0-10 [Provoked TS intensity]	30 days [n.r.]
Toffol, 2017	Parallel [University]	25.3 ± 6.7 [18-n.r.]	40 [35]	C2/Incisor & Canine	I: HP 35%[g] [20]<br>II: Light[b] [20]	I: 1 x 45' [3] - 7<br>II: no BG	I: no light<br>II: 20 x 30" (1') [3] - 7	405 ± 10 nm /n.r./n.r.	Spectrophotometer[f] [ΔE^*_{ab}] and shade guides[d] [ΔSGU]	VAS 0-10 [Spontaneous TS risk and intensity]	30 days [0]
Trevisan, 2022	Parallel [University]	23.2 ± n.r. [18-n.r.]	20 [81]	n.r./incisor & canine	II: HP 6%[q] [10]<br>III: HP 6%[q] + Light[k] [10]	I: 3 x 16' [3] - 7<br>II: 3 x 16' [3] - 7	I: no light<br>II: no light<br>III: 8 x 1' (60") [n.r.] - 7	405 ± 10/600 /n.r.	Spectrophotometer[l] [ΔE^*_{ab}]	VAS 0-10 [Spontaneous, Stimuli-induced TS risk and intensity]	21days [6]

						III: 3 x 16' [3] - 7					
Zanin, 2025	Parallel [University]	22.9 ± 5.7 [18-50]	84 [38]	A2/Canine	I: HP 35%[r] [28]<br>II: Light[b] [28]<br>III: HP 35%[r] + Light[b] [28]	I: 1 x 30' [3] - 7<br>II: no BG<br>III: 1 x 30' [3] - 7	I: no light<br>II: 20 x 1' (30'') [3] - 7<br>III: 20 x 1' (30'') [3] - 7	405 nm /n.r. /1.2 W	Spectrophotometer[f] [ΔE^*_{ab}] and shade guide[d] [ΔSGU]	VAS 0-10 [Spontaneous TS risk and intensity]	30 days [7]

a) 35% CP with sodium fluoride and potassium nitrate - Fórmula & Ação, São Paulo, SP, Brazil;
b) Bright Max Whitening BMW, MMOptics, São Carlos, SP, Brazil;
c) VITA Easyshade, Vident, Brea, CA;
d) Vita Classical, Vita Zahnfabrik, Bad Säckingen, Germany;
e) Nano White, DMC, São Carlos, SP, Brazil;
f) VITA Easyshade Advance 4.0 spectrophotometer, Vita Zahnfabrik, Bad Säckingen, Germany;
g) Whiteness HP, FGM, Joinvile, SC, Brazil;
h) Lase Peroxide HP 35%, DMC;
i) PowerBleaching Office Whitening®, BM4, Maringá, PR, Brazil;
j) Nano White Flex, DMC;
k) Whitening Plus, DMC;
l) VITA Easyshade V, Vita Zahnfabrik;
m) Whiteness HP Blue, FGM;
n) Colorimeter – Pocket Spec Technologies, Denver, CO, USA;
o) Glycerin Placebo;
p) 35% CP Whiteform, Formula & Ação;
q) Lase Peroxide HP 6%, DMC;
r) Clàriant Angelus Office 35, Angelus, Londrina, PR, Brazil)

Abbreviatons: ID: identification; SD: standard deviation; # número; n.r.: not reported; HP: hydrogen peroxide; CP: carbamide peroxide; BG: bleaching gel; SGU: shade guide units; VAS: visual analogue scale; TS: tooth sensitivity.

Supplementary Table 3. Model fit of all NMA.

**Full dataset**				
**Model fit parameters**	**ΔE^*_{ab}**	**ΔSGU/final SGU**	**Risk of TS**	**Intensity of TS**
Dbar	27.516	24.615	36.086	29.456
pD	23.666	22.940	20.170	28.843
DIC	51.182	47.555	56.255	58.299
Data points	28.000	25.000	31.000	25.000
**Subset of dataset excluding studies with high RoB**				
**Model fit parameters**	**ΔE^*_{ab}**	**ΔSGU/final SGU**	**Risk of TS**	**Intensity of TS**
Dbar	15.224	12.819	14.088	13.587
pD	14.410	12.137	10.199	12.067
DIC	29.633	24.955	24.288	25.655
Data points	16.000	13.000	13.000	13.000

Abbreviations: Mean deviance (Dbar); Number of parameters (pD); Deviance Information Criterion (DIC); Tooth sensitivity (TS).

Supplementary Table 4a. Directly estimate the certainty of the rating process for color change in ΔE^*_{ab}.

Comparison		Direct estimate									
		Absolute estimate (mean difference)			GRADE						
**Treatment 1**	**Treatment 2**	Point estimate	CI lower limit	CI upper limit	Risk of bias	Inconsistency	Indirectness	Publication bias	Rating for NMA	Imprecision	Final rating
VIOL	BG	2.97	1.05	4.90	Not serious[a]	Not serious[c]	Not serious[d]	Not serious[e]	HIGH	Serious[g]	MODERATE
BGVIOL	BG	-2.92	-3.98	1.85	Not serious[a]	Not serious[b]	Not serious[d]	Not serious[e]	HIGH	Serious[f]	MODERATE
VIOL	BGVIOL	6.52	3.69	9.36	Not serious[a]	Not serious[b]	Not serious[d]	Not serious[e]	HIGH	Not serious[h]	HIGH

Abbreviations
CI: confidence interval; **BG:** bleaching gel; **VIOL:** violet light; **BGVIOL:** combined bleaching gel + violet light; **NA:** not applicable; **NMA:** network-meta-analysis.

Minimally important difference (MID): 2.7 (acceptability threshold)

GRADE Working Group grades of evidence
High certainty: We are very confident that the true effect lies close to that of the estimate of the effect.
Moderate certainty: We are moderately confident in the effect estimate: The true effect is likely to be close to the estimate of the effect, but there is a possibility that it is substantially different.
Low certainty: Our confidence in the effect estimate is limited: The true effect may be substantially different from the estimate of the effect.
Very low certainty: We have very little confidence in the effect estimate: The true effect is likely to be substantially different from the estimate of effect.

Explanatory Footnotes
[a]Studies that carried large weight for the overall effect estimate rated as low risk of bias (not downgraded).
[b]There is substantial heterogeneity, but the inconsistency involves the magnitude rather than the direction of the effect (not downgraded).
[c]The substantial heterogeneity is largely attributed to two studies with a conflicting effect direction, but they carry small weights (not downgraded)
[d]There was no substantial difference between the identified evidence and the PICO question of the review regarding: patients, interventions, comparisons, outcomes.
[e]Comprehensiveness of the search strategies and methods to identify all available evidence.
[f]The CI crosses the MID threshold for benefit (downgraded by one level due to imprecision).
[g]The CI crosses the MID threshold for harm (downgraded by one level due to imprecision).
[h]The point estimate shows a large effect and plausible and the Crl does not cross MID threshold (not downgraded).

Supplementary Table 4b. Indirect estimate certainty of the rating process for color change in ΔE^*_{ab}.

Comparison		Indirect estimate												
		**Absolute estimate (mean difference)**			**GRADE**									
**Treatment 1**	**Treatment 2**	Point estimate	CrI lower limit	CrI upper limit	Common comparator	Treatment 1 vs. common comparator	Middle comparator	Common comparator vs. middle	Treatment 2 vs. common comparator	Start rating	Intransitivity	Rating for NMA	Imprecision	Final rating
VIOL	BG	NA	NA	NA	BGVIOL[1]	NA	NA	NA	NA	NA	NA	NA	NA	NA
BGVIOL	BG	NA	NA	NA	VIOL[1]	NA	NA	NA	NA	NA	NA	NA	NA	NA
VIOL	BGVIOL	3.77	-3.55	10.6	BG	HIGH			HIGH	HIGH	Not serious[a]	HIGH	Very serious[b]	LOW

Abbreviations
Crl: credible interval; **BG:** bleaching gel; **VIOL:** violet light; **BGVIOL:** combined bleaching gel + violet light; **NA:** not applicable; **NMA:** network-meta-analysis.

Minimally important difference (MID): 2.7 (acceptability threshold)

GRADE Working Group grades of evidence
High certainty: We are very confident that the true effect lies close to that of the estimate of the effect.
Moderate certainty: We are moderately confident in the effect estimate: The true effect is likely to be close to the estimate of the effect, but there is a possibility that it is substantially different.
Low certainty: Our confidence in the effect estimate is limited: The true effect may be substantially different from the estimate of the effect.
Very low certainty: We have very little confidence in the effect estimate: The true effect is likely to be substantially different from the estimate of effect.

Explanatory Footnotes
[1]Closed loop is made entirely by multi-armed trials.
[a]There was no concerns about the credibility of the effect estimate due to a potential mismatch between PICO characteristics.
[b]The Crl crosses the MID threshold for harm and benefit (downgraded by two levels due to imprecision).

Supplementary Table 4c. NMA estimates certainty of the rating process for color change in ΔE^*_{ab}.

Comparison		Network estimate								
		Absolute estimate (mean difference)			GRADE					
**Treatment 1**	**Treatment 2**	Point estimate	CrI lower limit	CrI upper limit	Starting evidence	Starting rating	Incoherence	Imprecision	Final rating	Best estimate
VIOL	BG	3.02	1.11	4.84	DIRECT	HIGH	None	Serious[b]	MODERATE	NMA
BGVIOL	BG	-3.13	-4.97	-1.33	DIRECT	HIGH	None	Serious[c]	MODERATE	NMA
VIOL	BGVIOL	6.16	4.08	8.18	DIRECT	HIGH	Not serious[a]	Not serious[d]	HIGH	NMA

Abbreviations
Crl: credible interval; **BG:** bleaching gel; **VIOL:** violet light; **BGVIOL:** combined bleaching gel + violet light; **NA:** not applicable; **NMA:** network-meta-analysis.

Minimally important difference (MID): 2.7 (acceptability threshold)

GRADE Working Group grades of evidence
High certainty: We are very confident that the true effect lies close to that of the estimate of the effect.
Moderate certainty: We are moderately confident in the effect estimate: The true effect is likely to be close to the estimate of the effect, but there is a possibility that it is substantially different.
Low certainty: Our confidence in the effect estimate is limited: The true effect may be substantially different from the estimate of the effect.
Very low certainty: We have very little confidence in the effect estimate: The true effect is likely to be substantially different from the estimate of effect.

Explanatory Footnotes
[a]Similar effect estimates among direct and indirect evidence, overlap of 95% Crl and nonsignificant p-value (not downgraded).
[b]The Crl crosses the MID threshold for harm (downgraded by one level due to imprecision).
[c]The Crl crosses the MID threshold for benefit (downgraded by one level due to imprecision).
[d]The point estimate shows a large effect and plausible and the Crl does not cross MID threshold (not downgraded).

Supplementary Table 5a. Directly estimate the certainty of the rating process for color change in ΔSGU/ final SGU.

Comparison		Direct estimate									
		Absolute estimate (mean difference)			GRADE						
**Treatment 1**	**Treatment 2**	Point estimate	CI lower limit	CI upper limit	Risk of bias	Inconsistency	Indirectness	Publication bias	Rating for NMA	Imprecision	Final rating
VIOL	BG	-3.68	2.00	5.36	Not serious[a]	Not serious[c]	Not serious[e]	Not serious[f]	HIGH	Not serious[h]	HIGH
BGVIOL	BG	-0.86	-2.35	0.63	Serious[b]	Serious[d]	Not serious[e]	Not serious[f]	LOW	Serious[g]	VERY LOW
VIOL	BGVIOL	3.85	2.26	5.44	Not serious[a]	Not serious[c]	Not serious[e]	Not serious[f]	HIGH	Not serious[h]	HIGH

Abbreviations
CI: confidence interval; **BG:** bleaching gel; **VIOL:** violet light; **BGVIOL:** combined bleaching gel + violet light; **NA:** not applicable; **NMA:** network-meta-analysis.

Minimally important difference (MID): 2 SGU

GRADE Working Group grades of evidence
High certainty: We are very confident that the true effect lies close to that of the estimate of the effect.
Moderate certainty: We are moderately confident in the effect estimate: The true effect is likely to be close to the estimate of the effect, but there is a possibility that it is substantially different.
Low certainty: Our confidence in the effect estimate is limited: The true effect may be substantially different from the estimate of the effect.
Very low certainty: We have very little confidence in the effect estimate: The true effect is likely to be substantially different from the estimate of effect.

Explanatory Footnotes
[a]Studies that carried large weight for the overall effect estimate rated as low risk of bias (not downgraded).
[b]Studies that carried large weight for the overall effect estimate rated as moderate/high risk of bias (downgraded by one level).
[c]There is substantial heterogeneity, but the inconsistency involves the magnitude rather than the direction of the effect (not downgraded).
[d]Different effect estimates among different studies, nonoverlap of 95% CI, high I^2, and significant p-value (downgraded by one level).
[e]There was no substantial difference between the identified evidence and the PICO question of the review regarding: patients, interventions, comparisons, outcomes.
[f]Comprehensiveness of the search strategies and methods to identify all available evidence
[g]The Crl crosses the MID threshold for benefit (downgraded by one level due to imprecision).
[h]The effect size is modest and plausible and the CI does not cross MID threshold (not downgraded due imprecision).

Supplementary Table 5b. Indirect estimate certainty of the rating process for color change in ΔSGU/ final SGU.

**Comparison**		**Indirect estimate**												
		**Absolute estimate (mean difference)**			**GRADE**									
**Treatment 1**	**Treatment 2**	Point estimate	CrI lower limit	CrI upper limit	Common comparator	Treatment 1 vs. common comparator	Middle comparator	Common comparator. vs. middle	Treatment 2 vs. common comparator	Start rating	Intransitivity	Rating for NMA	Imprecision	Final rating
VIOL	BG	NA	NA	NA	BGVIOL[1]	NA	NA	NA	NA	NA	NA	NA	NA	NA
BGVIOL	BG	NA	NA	NA	VIOL[1]	NA	NA	NA	NA	NA	NA	NA	NA	NA
VIOL	BGVIOL	5.70	1.04	10.5	BG	HIGH			LOW	LOW	Not serious[a]	LOW	Very serious[c]	VERY LOW

Abbreviations
Crl: credible interval; **BG:** bleaching gel; **VIOL:** violet light; **BGVIOL:** combined bleaching gel + violet light; **NA:** not applicable; **NMA:** network-meta-analysis.

Minimally important difference (MID): 2 SGU

GRADE Working Group grades of evidence
High certainty: We are very confident that the true effect lies close to that of the estimate of the effect.
Moderate certainty: We are moderately confident in the effect estimate: The true effect is likely to be close to the estimate of the effect, but there is a possibility that it is substantially different.
Low certainty: Our confidence in the effect estimate is limited: The true effect may be substantially different from the estimate of the effect.
Very low certainty: We have very little confidence in the effect estimate: The true effect is likely to be substantially different from the estimate of effect.

Explanatory Footnotes
[1]Closed loop is made entirely by multi-armed trials.
[a]There was no concerns about the credibility of the effect estimate due to a potential mismatch between PICO characteristics.
[b]The true effect is trivial, as the Crl falls within the two MID thresholds (not downgraded).
[c]The Crl includes the possibility of a trivial effect, but also includes the possibility of an important effect (downgraded by two levels due to imprecision).

Supplementary Table 5c. NMA estimates certainty of the rating process for color change in ΔSGU/ final SGU.

Comparison		Network estimate								
		Absolute estimate (mean difference)			GRADE					
**Treatment 1**	**Treatment 2**	Point estimate	CrI lower limit	CrI upper limit	Starting evidence	Starting rating	Incoherence	Imprecision	Final rating	Best estimate
VIOL	BG	3.37	1.58	5.19	DIRECT	HIGH	None	Serious[c]	MODERATE	DIRECT
BGVIOL	BG	-0.85	-2.59	0.84	DIRECT	LOW	None	Serious[d]	VERY LOW	NMA
VIOL	BGVIOL	4.22	2.22	6.31	DIRECT	HIGH	Not serious[a]	Not serious[b]	HIGH	NMA

Abbreviations
Crl: credible interval; **BG:** bleaching gel; **VIOL:** violet light; **BGVIOL:** combined bleaching gel + violet light; **NA:** not applicable; **NMA:** network-meta-analysis.

Minimally important difference (MID): 2 SGU

GRADE Working Group grades of evidence
High certainty: We are very confident that the true effect lies close to that of the estimate of the effect.
Moderate certainty: We are moderately confident in the effect estimate: The true effect is likely to be close to the estimate of the effect, but there is a possibility that it is substantially different.
Low certainty: Our confidence in the effect estimate is limited: The true effect may be substantially different from the estimate of the effect.
Very low certainty: We have very little confidence in the effect estimate: The true effect is likely to be substantially different from the estimate of effect.

Explanatory Footnotes
[a]Similar effect estimates among direct and indirect evidence, overlap of 95% Crl and nonsignificant p-value (not downgraded).
[b]The effect size is modest and plausible and the Crl does not cross MID threshold (not downgraded due imprecision).
[c]The Crl crosses the MID threshold for harm (downgraded by one level due to imprecision).
[d]The Crl crosses the MID threshold for benefit (downgraded by one level due to imprecision).

Supplementary Table 6a. Directly estimate the certainty of the rating process for the risk of tooth sensitivity.

Comparison		Direct estimate													
		Relative estimate (risk ratio)			Absolute estimate (per 1000)			GRADE							
**Treatment 1**	**Treatment 2**	Point estimate	CI lower limit	CI upper limit	Point estimate	CI lower limit	CI upper limit	Risk of bias	Inconsistency	Indirectness	Publication bias	Rating for NMA	Imprecision	Final rating
VIOL	BG	0.27	0.10	0.70	-480	-592	-197	Very serious[b]	Serious[d]	Not serious[f]	Not serious[g]	VERY LOW	Not serious[i]	VERY LOW
BGVIOL	BG	1.03	0.79	1.34	15	-104	168	Serious[a]	Serious[e]	Not serious[f]	Not serious[g]	LOW	Serious[h]	VERY LOW
VIOL	BGVIOL	0.32	0.11	0.88	-439	-575	-77	Serious[a]	Not serious[c]	Not serious[f]	Not serious[g]	MODERATE	Serious[j]	LOW

Abbreviations
CI: confidence interval; **BG:** bleaching gel; **VIOL:** violet light; **BGVIOL:** combined bleaching gel + violet light; **NA:** not applicable; **NMA:** network-meta-analysis.

Minimally important difference (MID): 100 patients per 1000

GRADE Working Group grades of evidence
High certainty: We are very confident that the true effect lies close to that of the estimate of the effect.
Moderate certainty: We are moderately confident in the effect estimate: The true effect is likely to be close to the estimate of the effect, but there is a possibility that it is substantially different.
Low certainty: Our confidence in the effect estimate is limited: The true effect may be substantially different from the estimate of the effect.
Very low certainty: We have very little confidence in the effect estimate: The true effect is likely to be substantially different from the estimate of effect.

Explanatory Footnotes
[a]Studies that carried large weight for the overall effect estimate rated as moderate risk of bias (downgraded by one level).
[b]Studies that carried large weight for the overall effect estimate rated as high risk of bias (downgraded by two levels).
[c]There is substantial heterogeneity, but the inconsistency involves the magnitude rather than the direction of the effect (not downgraded).
[d]The substantial heterogeneity is largely attributed to one study with a conflicting effect direction, which carries a large weight (downgraded by one level)
[e]Different effect estimates among different studies, nonoverlap of 95% CI, high I^2, and significant p-value (downgraded by one level).
[f]There was no substantial difference between the identified evidence and the PICO question of the review regarding: patients, interventions, comparisons or outcomes.
[g]Comprehensiveness of the search strategies and methods to identify all available evidence.
[h]The CI crosses the MID threshold for harm and benefit (downgraded by one level due to imprecision).
[i]The effect size is modest and plausible and the CI does not cross MID threshold (not downgraded due imprecision).
[j]The CI crosses the MID threshold for benefit (downgraded by one level due to imprecision).

Supplementary Table 6b. Indirect estimate certainty of the rating process for the risk of tooth sensitivity.

Comparison		Indirect estimate															
		Relative estimate (risk ratio)			Absolute estimate (per 1000)			GRADE									
**Treatment 1**	**Treatment 2**	Point estimate	CrI lower limit	CrI upper limit	Point estimate	CI lower limit	CI upper limit	Common comparator	Treatment 1 vs. common comparator	Middle comparator	Common comparator. vs. middle	Treatment 2 vs. common comparator	Start rating	Intransitivity	Rating for NMA	Imprecision	Final rating
VIOL	BG	NA	NA	NA	NA	NA	NA	BGVIOL[1]	NA	NA	NA	NA	NA	NA	NA	NA	NA
BGVIOL	BG	NA	NA	NA	NA	NA	NA	VIOL[1]	NA	NA	NA	NA	NA	NA	NA	NA	NA
VIOL	BGVIOL	0.16	0.01	1.4	-427	-501	102	BG	VERY LOW			LOW	VERY LOW	Not serious[a]	VERY LOW	Very serious[b]	VERY LOW

Abbreviations
Crl: credible interval; **BG:** bleaching gel; **VIOL:** violet light; **BGVIOL:** combined bleaching gel + violet light; **NA:** not applicable; **NMA:** network-meta-analysis.

Minimally important difference (MID): 100 patients per 1000

GRADE Working Group grades of evidence
High certainty: We are very confident that the true effect lies close to that of the estimate of the effect.
Moderate certainty: We are moderately confident in the effect estimate: The true effect is likely to be close to the estimate of the effect, but there is a possibility that it is substantially different.
Low certainty: Our confidence in the effect estimate is limited: The true effect may be substantially different from the estimate of the effect.
Very low certainty: We have very little confidence in the effect estimate: The true effect is likely to be substantially different from the estimate of effect.

Explanatory Footnotes
[1]Closed loop is made entirely by multi-armed trials.
[a]There was no concerns about the credibility of the effect estimate due to a potential mismatch between PICO characteristics.
[b]The CI crosses the MID threshold for harm and benefit (downgraded by two levels due to imprecision).

Supplementary Table 6c. NMA estimates certainty of the rating process for the risk of tooth sensitivity.

Comparison		Network estimate											
		**Relative estimate (risk ratio)**			**Absolute estimate (per 1000)**			**GRADE**					
**Treatment 1**	**Treatment 2**	Point estimate	CrI lower limit	CrI upper limit	Point estimate	CI lower limit	CI upper limit	Starting evidence	Starting rating	Incoherence	Imprecision	Final rating	Best estimate
VIOL	BG	0.34	0.16	0.63	-326	-415	-183	DIRECT	VERY LOW	None	Not serious[c]	VERY LOW	NMA
BGVIOL	BG	1.03	0.65	1.79	20	-168	380	DIRECT	LOW	None	Very serious[b]	VERY LOW	NMA
VIOL	BGVIOL	0.33	0.15	0.62	-326	-423	-183	DIRECT	MODERATE	Not serious[a]	Not serious[c]	MODERATE	NMA

Abbreviations
Crl: credible interval; **BG:** bleaching gel; **VIOL:** violet light; **BGVIOL:** combined bleaching gel + violet light; **NMA:** network-meta-analysis.

Minimally important difference (MID): 100 patients per 1000

GRADE Working Group grades of evidence
High certainty: We are very confident that the true effect lies close to that of the estimate of the effect.
Moderate certainty: We are moderately confident in the effect estimate: The true effect is likely to be close to the estimate of the effect, but there is a possibility that it is substantially different.
Low certainty: Our confidence in the effect estimate is limited: The true effect may be substantially different from the estimate of the effect.
Very low certainty: We have very little confidence in the effect estimate: The true effect is likely to be substantially different from the estimate of effect.

Explanatory Footnotes
[a]Similar effect estimates among direct and indirect evidence, overlap of 95% Crl and nonsignificant p-value (not downgraded).
[b]The CI crosses the MID threshold for harm and benefit (downgraded by two levels due to imprecision).
[c]The effect size is modest and plausible and the Crl does not cross MID threshold (not downgraded due imprecision).

Supplementary Table 7a. Directly estimate the certainty of the rating process for the intensity of tooth sensitivity (VAS 0-10).

**Comparison**		**Direct estimate**									
		**Absolute estimate (mean difference)**			**GRADE**						
**Treatment 1**	**Treatment 2**	Point estimate	CI lower limit	CI upper limit	Risk of bias	Inconsistency	Indirectness	Publication bias	Rating for NMA	Imprecision	Final rating
VIOL	BG	-2.81	-5.70	0.08	Serious[a]	Not serious[c]	Not serious[e]	Not serious[f]	MODERATE	Serious[h]	LOW
BGVIOL	BG	-0.16	-0.77	0.46	Very serious[b]	Serious[d]	Not serious[e]	Not serious[f]	VERY LOW	Not serious[g]	VERY LOW
VIOL	BGVIOL	-2.45	-3.99	-0.92	Serious[a]	Not serious[c]	Not serious[e]	Not serious[f]	MODERATE	Serious[h]	LOW

Abbreviations
CI: confidence interval; **BG:** bleaching gel; **VIOL:** violet light; **BGVIOL:** combined bleaching gel + violet light; **NA:** not applicable; **NMA:** network-meta-analysis.

Minimally important difference (MID): 2 units

GRADE Working Group grades of evidence
High certainty: We are very confident that the true effect lies close to that of the estimate of the effect.
Moderate certainty: We are moderately confident in the effect estimate: The true effect is likely to be close to the estimate of the effect, but there is a possibility that it is substantially different.
Low certainty: Our confidence in the effect estimate is limited: The true effect may be substantially different from the estimate of the effect.
Very low certainty: We have very little confidence in the effect estimate: The true effect is likely to be substantially different from the estimate of effect.

Explanatory Footnotes
[a]Studies that carried large weight for the overall effect estimate rated as moderate risk of bias (downgraded by one level).
[b]Studies that carried large weight for the overall effect estimate rated as high risk of bias (downgraded by two levels).
[c]There is substantial heterogeneity, but the inconsistency involves the magnitude rather than the direction of the effect (not downgraded).
[d]Different effect estimates among different studies, nonoverlap of 95% CI, high I^2, and significant p-value (downgraded by one level).
[e]There was no substantial difference between the identified evidence and the PICO question of the review regarding: patients, interventions, comparisons, outcomes.
[f]Comprehensiveness of the search strategies and methods to identify all available evidence.
[g]The true effect is trivial, as the CI falls within the two MID thresholds (not downgraded due imprecision).
[h]The CI crosses the MID threshold for benefit (downgraded by one level due to imprecision).

Supplementary Table 7b. Indirect estimate certainty of the rating process for intensity of tooth sensitivity (VAS 0-10).

**Comparison**		**Indirect estimate**												
		**Absolute estimate (mean difference)**			**GRADE**									
**Treatment 1**	**Treatment 2**	Point estimate	CrI lower limit	CrI upper limit	Common comparator	Treatment 1 vs. common comparator	Middle comparator	Common comparator. vs. middle	Treatment 2 vs. common comparator	Start rating	Intransitivity	Rating for NMA	Imprecision	Final rating
VIOL	BG	NA	NA	NA	BGVIOL[1]	NA	NA	NA	NA	NA	NA	NA	NA	NA
BGVIOL	BG	NA	NA	NA	VIOL[1]	NA	NA	NA	NA	NA	NA	NA	NA	NA
VIOL	BGVIOL	-1.66	-8.81	5.51	BG	MODERATE			VERY LOW	VERY LOW	Not serious[a]	VERY LOW	Very serious[b]	VERY LOW

Abbreviations
Abbreviations
Crl: credible interval; **BG:** bleaching gel; **VIOL:** violet light; **BGVIOL:** combined bleaching gel + violet light; **NA:** not applicable; **NMA:** network-meta-analysis.

Minimally important difference (MID): 2 units

GRADE Working Group grades of evidence
High certainty: We are very confident that the true effect lies close to that of the estimate of the effect.
Moderate certainty: We are moderately confident in the effect estimate: The true effect is likely to be close to the estimate of the effect, but there is a possibility that it is substantially different.
Low certainty: Our confidence in the effect estimate is limited: The true effect may be substantially different from the estimate of the effect.
Very low certainty: We have very little confidence in the effect estimate: The true effect is likely to be substantially different from the estimate of effect.

Explanatory Footnotes
[1]Closed loop is made entirely by multi-armed trials.
[a]There was no concerns about the credibility of the effect estimate due to a potential mismatch between PICO characteristics.
[b]The Crl includes the possibility of an important effect for harm and benefit (downgraded by two levels due to imprecision).

Supplementary Table 7c. NMA estimates certainty of the rating process for intensity of tooth sensitivity (VAS 0-10).

**Comparison**		**Network estimate**								
		**Absolute estimate (mean difference)**			**GRADE**					
**Treatment 1**	**Treatment 2**	Point estimate	CrI lower limit	CrI upper limit	Starting evidence	Starting rating	Incoherence	Imprecision	Final rating	Best estimate
VIOL	BG	-2.87	-4.83	-0.96	DIRECT	MODERATE	None	Serious[c]	LOW	NMA
BGVIOL	BG	-0.24	-1.93	1.42	DIRECT	VERY LOW	None	Not serious[b]	VERY LOW	NMA
VIOL	BGVIOL	-2.53	-4.63	-0.59	DIRECT	MODERATE	Not serious[a]	Serious[c]	LOW	NMA

Abbreviations
Crl: credible interval; **BG:** bleaching gel; **VIOL:** violet light; **BGVIOL:** combined bleaching gel + violet light; **NMA:** network-meta-analysis.

Minimally important difference (MID): 2 units

GRADE Working Group grades of evidence
High certainty: We are very confident that the true effect lies close to that of the estimate of the effect.
Moderate certainty: We are moderately confident in the effect estimate: The true effect is likely to be close to the estimate of the effect, but there is a possibility that it is substantially different.
Low certainty: Our confidence in the effect estimate is limited: The true effect may be substantially different from the estimate of the effect.
Very low certainty: We have very little confidence in the effect estimate: The true effect is likely to be substantially different from the estimate of effect.

Explanatory Footnotes
[a]Similar effect estimates among direct and indirect evidence, overlap of 95% Crl and nonsignificant p-value (not downgraded).
[b]The true effect is trivial, as the Crl falls within the two MID thresholds (not downgraded).
[c]Crl crosses the MID threshold for benefit (downgraded by one level due to imprecision).
